# Synergistic tailoring of structural, optical, dielectric, and magnetic responses in Ce/Ni- and Nd/Ni-co-doped M-type BaFe_12_O_19_ nano-compositions

**DOI:** 10.1039/d6ra03457h

**Published:** 2026-08-10

**Authors:** Mohammed Ahmed Wahba

**Affiliations:** a Inorganic Chemistry Department, National Research Centre (NRC) El Buhouth St., Dokki Cairo 12622 Egypt mohamedwahba12@gmail.com

## Abstract

M-type BaFe_12_O_19_ is a technologically important ferrite owing to its large uniaxial anisotropy, chemical robustness, and relevance to magnetic and high-frequency devices. Here, pristine BaFe_12_O_19_ (BF), Ba_0.97_Ce_0.03_Fe_11.97_Ni_0.03_O_19_ (CNBF), and Ba_0.97_Nd_0.03_Fe_11.97_Ni_0.03_O_19_ (NNBF) nanocompositions were synthesized by a sol–gel route to probe the effect of Ni/rare-earth co-substitution on the coupled structural, optical, dielectric, and magnetic responses. Rietveld-refined X-ray diffraction data confirmed retention of the magnetoplumbite structure in all samples, with a slight lattice contraction upon co-substitution, most evident for NNBF. Microstructural analysis revealed a denser and more homogeneous morphology for CNBF, whereas NNBF showed greater heterogeneity, consistent with stronger local structural perturbation. Optical measurements showed that the band gap changed from 1.647 eV for BF to 1.652 eV for CNBF, while a lower value of 1.631 eV was obtained for NNBF; similarly, the Urbach energy results suggested lower disorder in CNBF and more pronounced localized states in NNBF. A pronounced suppression of the low-frequency dielectric constant was observed, decreasing from ∼2750 in BF to ∼1230 in CNBF and ∼1080 in NNBF, accompanied by lower dielectric loss, indicative of reduced interfacial polarization and improved charge compensation. Magnetically, co-substitution markedly enhanced the saturation magnetization from 28.04 emu g^−1^ (BF) to 36.75 emu g^−1^ (CNBF) and 36.41 emu g^−1^ (NNBF), while reducing the coercive field from 3361 Oe to 3008 and 2804 Oe, respectively. The maximum energy product also increased to 2.81 × 10^5^ erg g^−1^ for CNBF. These results show that Ni/rare-earth co-substitution is a suitable route to adjust the multifunctional performance of BaFe_12_O_19_ with the Ce/Ni co-doping providing the best overall response among the studied compositions.

## Abstract 2

Ce/Ni and Nd/Ni co-substitution in BaFe_12_O_19_ nanocompositions enhances saturation magnetization and tunes the optical bandgap. Structural analysis confirms lattice contraction and dense CNBF morphology, with suppressed interfacial polarization.

## Graphical abstract

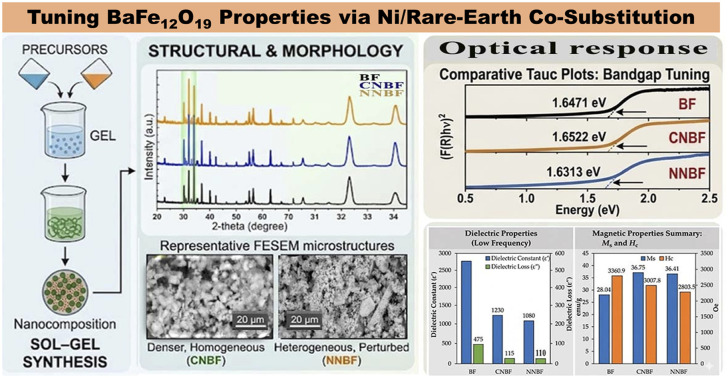

## 1 Introduction

Hexagonal M-type barium hexaferrite (BaFe_12_O_19_) is a material of considerable scientific and technological interest due to its favorable magnetic characteristics, including high saturation magnetization (*M*_s_), pronounced magnetocrystalline anisotropy, elevated Curie temperature (*T*_c_) and excellent chemical stability.^1,2^ These characteristics make it a versatile platform for various industrial applications, ranging from high-density magnetic storage and high-coercivity permanent magnets to advanced microwave components, EMI shielding, and high-frequency devices.^3,4^ The broader scientific and technological importance of hexagonal ferrites, including their structural diversity, synthesis routes, magnetic behavior, and applications in permanent magnets, data storage, and microwave/GHz devices, has been comprehensively reviewed by Pullar.^5^ BaFe_12_O_19_ crystallizes in the magnetoplumbite-type hexagonal structure with the *P*6(3)/*mmc* space group. In this framework, Fe^3+^ cations are distributed over five nonequivalent lattice positions, namely three octahedral sites (12k, 2a, and 4f_2_), one tetrahedral site (4f_1_), and one trigonal bipyramidal site (2b). The overall magnetic behavior of the material arises from the ferrimagnetic coupling among these sublattices, which determines the resultant magnetic moment, where ions at the 2a, 2b, and 12k sites exhibit up-spin alignments and those at the 4f_1_ and 4f_2_ sites show down-spin alignments.^6,7^

Despite its excellent intrinsic properties and long-standing technological importance, BaFe_12_O_19_ remains an active research platform because its functional performance is highly sensitive to synthesis route, dopant chemistry, cation distribution, and microstructure.^5^ However, the practical deployment of BaFe_12_O_19_ in advanced electronic and photonic systems is often limited by high dielectric loss at low frequencies and the strong interdependence between its optical, magnetic, and electrical responses.^8,9^ Rational strategies for overcoming these limitations typically involve elemental substitution to precisely tune the cation distribution, defect chemistry, and microstructure. Various metals and transition-metal ions (*e.g.*, Ga, Al, Cr, Co, Ti, *etc.*)^10–14^ and rare-earth ions (*e.g.*, Gd, La, Sm, and Ho)^5,15–17^ have been systematically introduced into the BaFe_12_O_19_ lattice to modify lattice parameters, superexchange interactions, and charge-carrier dynamics. Elemental substitution serves as a powerful strategy to tune intrinsic characteristics including (*M*_s_), (*H*_c_), magnetocrystalline anisotropy (*K*), and Curie temperature (*T*_c_). Research into mono-doping has highlighted the distinct functional roles of specific ions. For instance, substitution with aluminum Al^3+^ can significantly increase the coercive force, resulting in a magnetically harder material, whereas gallium (Ga^3+^) substitution typically leads to a decrease in magnetic parameters, making the material magnetically softer.^10,18^ The introduction of titanium (Ti^4+^) has been shown to improve dielectric properties at microwave frequencies and influence both saturation magnetization and the coercive field, enabling its use as a microwave absorber.^14^ Furthermore, substitution with rare-earth ions induces lattice distortions and alters spin dynamics due to their larger ionic radii compared to Fe^3+^, which directly impacts complex permeability and permittivity. Specific elements like holmium (Ho) have been reported to enhance coercivity,^19^ while scandium (Sc) can induce low-temperature spin glass-like phase transitions.^20^ La^3+^ doping in BaFe_12_O_19_ was also reported to improve crystallinity and increase crystallite size while gradually reducing the band gap, thus underscoring its potential for use in magnetic recording, electronics, permanent magnets, and communication devices.^21^

Building upon these mono-doping studies, co-doping with two different elements offers an effective route for simultaneously tuning multiple material parameters, including charge compensation, cation distribution, and microstructural evolution. For example, dual rare-earth substitution (*e.g.*, Dy/Ce) has been reported to combine high magnetic moments with electronic polarization and hopping transport extrinsic conduction, which synergistically enhances reflection loss and microwave absorption in the GHz region.^22^ Similarly, co-doping transition metals like nickel and copper (Ni/Cu) produce additional electronic species that contribute to conduction-based polarization, thereby yielding elevated permittivity magnitudes while maintaining the magnetic interactions necessary to enhance magnetization.^23^ Rare-earth/transition-metal co-doping strategies (*e.g.*, La/Gd or Ni/Gd) have been demonstrated that to be an effective route to simultaneously regulate magnetic anisotropy and electrical transport properties in BaFe_12_O_19_.^24,25^

The substitution of transition-metal or rare-earth in BaFe_12_O_19_ has been the subject of many studies. Nevertheless, direct comparative studies on the influence of different rare-earth/Ni co-substitution strategies on the coupled structural, optical, dielectric and magnetic responses under identical synthesis conditions are relatively scarce. In particular, a systematic comparison between Ce/Ni and Nd/Ni co-doping has not been fully addressed. So, the present work is dedicated to the development of structure–property relationships controlling these multifunctional responses and to elucidate how the selection of rare-earth dopant influences the general behavior of BaFe_12_O_19_. In this regard, the present work is aimed to understand the impact of the simultaneous substitution of Ni at Fe sites and Ce/Nd at Ba sites on the defect chemistry, lattice distortion and cation distribution of M-type BaFe_12_O_19_ and how these coupled changes control its magnetic, dielectric and optical behavior. Compared with previous reports on rare-earth- or transition-metal-substituted BaFe_12_O_19_, the present study provides a comparative structure–property analysis of BaFe_12_O_19_ co-doping with two closely related rare-earth/Ni. The dual substitution is suggested to induce the lattice perturbation *via* the coupled cation redistribution, defect compensation and anisotropic structural distortion, which affect the superexchange, charge-carrier hopping, grain-boundary polarization and band-edge disorder. To investigate these effects, Ni/Nd and Ni/Ce-co-doped BaFe_12_O_19_ nanocompositions were synthesized by the sol–gel method, which provides molecular-level homogeneity and relatively low-temperature crystallization helping to suppress impurity formation and control grain growth.^26–28^ The Ni^2+^ ion is expected to occupy the octahedral Fe sites which will affect the coercivity and hopping transport. The size and electronic features of Nd^3+^ and Ce species can cause local lattice deformation and change the Fe–O–Fe superexchange.^29–31^ Such a strategy allows for a systematic assessment of the subtle dopant-induced structural and defect-related changes leading to the multifunctional property variations in substituted BaFe_12_O_19_.

## 2 Experimental procedure

### 2.1 Materials

Reagent-grade [Ba(NO_33_)_2_], [Fe(NO_3_)_3_·9H_2_O], [Ni(NO_3_)_2_·6H_2_O], (Nd_2_O_3_), (NH_4_)_2_Ce(NO_3_)_6_, and citric acid (C_6_H_8_O_7_) were utilized as starting materials without further purification. Deionized water was used as the solvent throughout the synthesis.

### 2.2 Synthesis of BaFe_12_O_19_ and co-doped samples

Synthesis of undoped BaFe_12_O_19_ (BF), Nd/Ni co-doped Ba_0.97_Nd_0.03_Fe_11.97_Ni_0.03_O_19_, (NNBF), and Ce/Ni co-doped Ba_0.97_Ce_0.03_Fe_11.97_Ni_0.03_O_19_, (CNBF) nanocompositions were achieved through a sol–gel route. For synthesis of the undoped BF sample, separate aqueous solutions of Ba(NO_3_)_2_ (0.5227 g, 2.000 mmol) and Fe(NO_3_)_3_·9H_2_O (9.696 g, 24.000 mmol) were prepared in deionized water under continuous magnetic stirring and subsequently mixed to yield a homogeneous precursor solution. Citric acid (4.866 g, 25.333 mmol) was provided as a chelating agent, maintaining a total metal ion to citric acid molar ratio of 3 : 1 to complex the metal ions and promote molecular-level homogeneity. The citric acid to total metal ion molar ratio was maintained at an appropriate value to ensure complete complexation and stable gel formation. For the Nd/Ni co-doped sample NNBF with nominal composition Ba_0.97_Nd_0.03_Fe_11.97_Ni_0.03_O_19_, Ba(NO_3_)_2_ (0.507 g, 1.940 mmol), Nd_2_O_3_ (0.0101 g, 0.0300 mmol), Fe(NO_3_)_3_·9H_2_O (9.40512 g, 23.280 mmol), and Ni(NO_3_)_2_·6H_2_O (0.0209 g, 0.0719 mmol) were used. Nd_2_O_3_ was first dissolved in a small amount of dilute nitric acid under mild heating to form a clear nitrate solution before being added to the mixed precursor solution. The amount of Nd_2_O_3_ used corresponds to 0.0601 mmol of Nd^3+^ ions. Citric acid (4.742 g, 24.681 mmol) was then added, corresponding to a total metal ion to citric acid molar ratio of 3 : 1. For the Ce/Ni co-doped sample CNBF with nominal composition Ba_0.97_Ce_0.03_Fe_11.97_Ni_0.03_O_19_, Ba(NO_3_)_2_ (0.507 g, 1.940 mmol), (NH_4_)_2_Ce(NO_3_)_6_ (0.0329 g, 0.0600 mmol), Fe(NO_3_)_3_·9H_2_O (9.40512 g, 23.280 mmol), and Ni(NO_3_)_2_·6H_2_O (0.0209 g, 0.0719 mmol) were employed. The cerium precursor was solubilized directly in deionized H_2_O and then added to the main precursor solution. Citric acid (4.746 g, 24.701 mmol) was subsequently added as a chelating agent while maintaining a total metal ion to citric acid molar ratio of 3 : 1. After complete mixing of the precursors, aqueous ammonia was added dropwise until the solution pH reached 7. The resulting solutions were stirred continuously and heated until a clear and homogeneous sol was formed. Further evaporation of the solvent transformed the sol into a viscous gel. The gels were dried to obtain xerogel precursors, then ground into fine powders and subjected to calcination at 900 °C for 4 h to form the crystalline M-type hexaferrite phase. After calcination, the powders were cooled naturally to room temperature and gently reground for further characterization.

### 2.3 Characterization

The phase composition and crystal structure of the synthesized powders were examined by X-ray diffraction (XRD) PANalytical X'Pert PRO diffractometer; using Cu Kα radiation (*λ* = 1.5406 Å). The diffraction data were collected over an appropriate 2*θ* range to identify the crystalline phases and evaluate the effect of co-doping on the magnetoplumbite structure. Structural parameters such as lattice constants, unit-cell volume, and crystallite size were determined from the XRD data, and detailed structural refinement was performed using the Rietveld method. The morphology mapping analysis and microstructural features of the powders were investigated by scanning electron microscopy (SEM/EDx, model Quanta 250 FEG). Fourier transform infrared spectroscopy (FTIR) using a Brucker Vertex 80 V spectrometer in the range of 4000–400 cm^−1^ to analyze the characteristic vibrational modes and confirm the formation of the ferrite framework. Optical properties were studied by diffuse reflectance spectroscopy (DRS), JASCO V-570 spectrophotometer, in the wavelength range of 200–2500 nm. The optical band gap was estimated using the Kubelka–Munk function and Tauc plots, while the Urbach energy was obtained from the exponential absorption edge region. The dielectric properties, including the dielectric constant (*ε*′), dielectric loss (*ε*″), loss tangent (tan *δ*), and AC conductivity (*σ*_ac_), were measured as a function of frequency at room temperature using an LCR analyzer (Hitester, model Hioki 3532-50, manufactured in Japan). For electrical measurements, the powders were pressed into pellet form using a suitable uniaxial pressure. Magnetic hysteresis loops were recorded at room temperature using a vibrating sample magnetometer, VSM, Model LDJ 9600-1 to determine the saturation magnetization, remanent magnetization, coercivity, anisotropy-related parameters, and maximum energy product.

## 3 Results and discussion

### 3.1 XRD

**Fig. 1:**
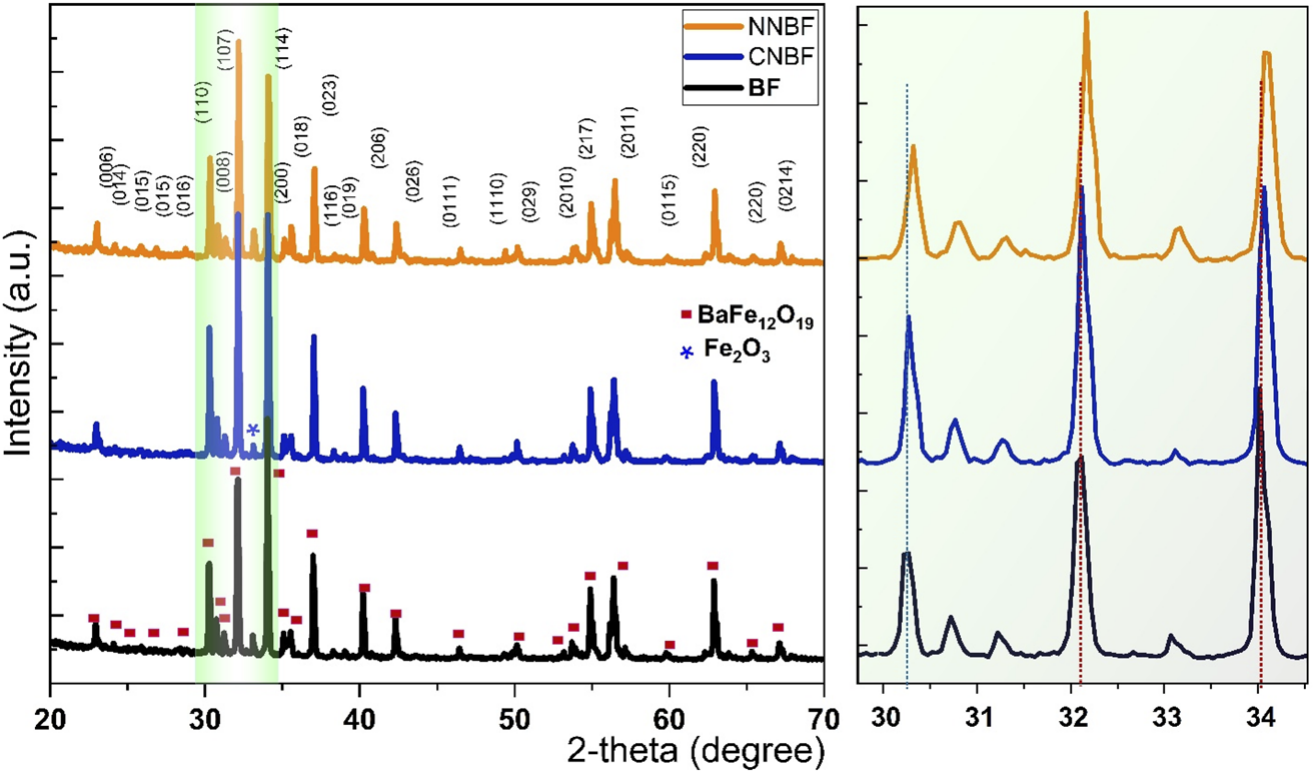
(left) XRD pattern of undoped, Ce/Ni, Nd/Ni co-doped BaFe_12_O_19_ nanocompostions; (right) 30–34.5 zoomed area shows shifts in (110) and (017) peaks.

The XRD patterns of the undoped BaFe_12_O_19_ (BF) and the co-doped samples, Ba_0.97_Nd_0.03_Fe_11.97_Ni_0.03_O_19_ (NNBF) and Ba_0.97_Ce_0.03_Fe_11.97_Ni_0.03_O_19_ (CNBF), synthesized by the sol–gel method (Fig. 1), indicate that the main crystalline phase in all samples is the magnetoplumbite hexaferrite structure of M-type BaFe_12_O_19_, indexed to the hexagonal system with space group *P*6(3)/*mmc* (COD No. 9008137).^32^ All diffraction maxima can be indexed to the standard diffraction pattern of BaFe_12_O_19_, revealing the successful development of the M-type structure after calcination. The characteristic diffraction peaks corresponding to planes such as (110), (107), (114), (200), (203), (206), (217), (2011), and (220) are clearly visible, showing that the basic hexaferrite framework is preserved after co-doping with Ni/Nd and Ni/Ce.

A comparison of the relative peak intensities shows that co-doping affects the crystallization behavior of specific planes. The intensity of the (114) reflection increases in the CNBF sample but decreases in the NNBF sample, whereas the intensities of the (107) and (110) reflections increase in both co-doped compositions relative to the undoped BF sample. These changes suggest that the introduction of Ni/Ce and Ni/Nd modifies the degree of crystallinity and preferred orientation, and may also be associated with changes in cation occupancy among 12k, 2a, 4f_1_, 4f_2_, and 2b sites of hexaferrite lattice.^33^ Since these crystallographic sites contribute differently to the structure factor of each reflection, even slight redistribution of cations can alter the relative intensity of particular peaks. In this context, the enhancement of the (107) and (110) peaks in both CNBF and NNBF may indicate improved ordering or preferential growth along these crystallographic directions after co-doping, while the opposite trend of the (114) peak in the two samples implies that Ce- and Nd-containing compositions affect the internal cation arrangement and microstructural development differently. In addition to the main hexaferrite phase, a minor secondary phase indexed to Fe_2_O_3_ is also detected in the diffractograms. Based on the figure, the Fe_2_O_3_ contribution is reduced in the CNBF sample and increased in the NNBF sample compared with the parent BF composition. This observation suggests that Ce/Ni co-doping favors better suppression of the iron oxide secondary phase, whereas the Nd/Ni co-doped sample retains a relatively higher amount of Fe_2_O_3_ residual. A closer examination of the enlarged XRD region 2*θ* (Fig. 1, right) shows that the principal reflections of the co-doped samples are slightly shifted relative to those of the undoped BF sample, providing clear evidence of a dopant-induced modification of the interplanar spacing in the BaFe_12_O_19_ lattice. Because all reflections can still be indexed to the hexagonal magnetoplumbite structure of M-type BaFe_12_O_19_ with space group, these peak displacements are attributed to lattice distortion associated with cation substitution rather than to any structural transformation of the parent phase. In M-type hexaferrite, Fe^3+^ ions occupy five crystallographically nonequivalent sublattices, namely 12k, 2a, 4f_1_, 4f_2_, and 2b, whereas Ba^2+^ occupies the large interlayer site between the spinel blocks. Consequently, substitution by Ni^2+^ and rare-earth ions such as Ce^4+^ and Nd^3+^ can perturb the local coordination environment, modify cation distribution among these sublattices, and generate internal strain within the magnetoplumbite framework. Although Ni^2+^ is slightly larger (0.690) than Fe^3+^ (0.645) in octahedral coordination,^34^ the overall structural response in the present system is dominated by the simultaneous replacement of Ba^2+^ by smaller rare-earth ions and by defect-assisted relaxation processes, which together result in a net contraction of the lattice. This contraction may also be promoted by local rearrangement of cations among the available Fe sites and by the structural accommodation required to maintain charge neutrality during co-doping.

The charge-compensation process can be rationalized using Kröger–Vink notation. Substitution of Ni^2+^ for Fe^3+^ introduces an acceptor defect according to:
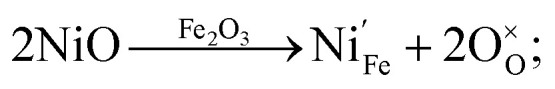
whereas substitution of Nd^3+^/Ce^4+^ for Ba^2+^ generates donor defects according to: 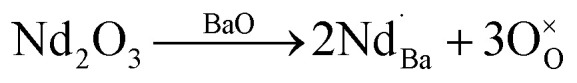

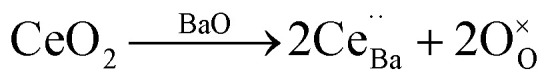


Under these conditions, internal donor–acceptor charge compensation may occur between rare-earth ions substituting at the Ba site and Ni ions substituting at the Fe site:
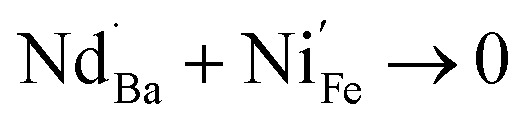

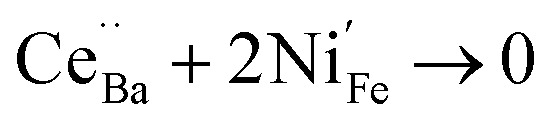


When this internal compensation is not locally complete, the lattice may accommodate the residual charge imbalance through defect formation, particularly oxygen vacancies, as represented by:
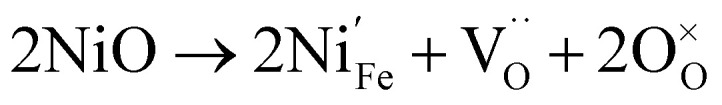


These charge-compensation mechanisms can modify local bond lengths, induce lattice strain, and influence cation distribution among the crystallographic sublattices, thereby contributing directly to the slight but measurable shifts observed in the diffraction peaks.

**Fig. 2:**
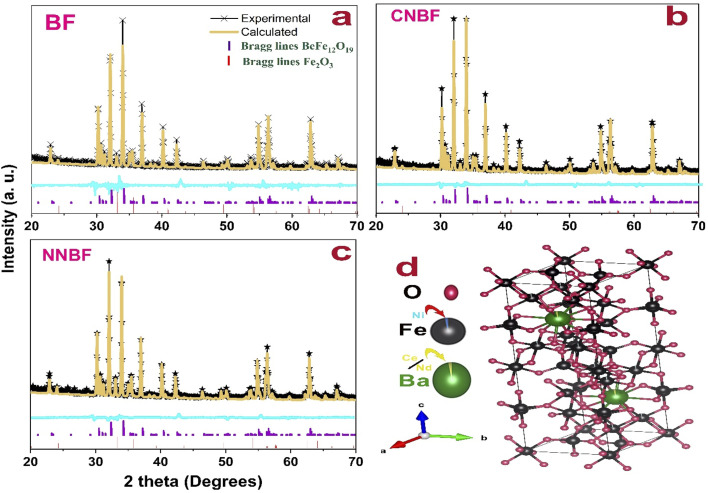
(a–c) Rietveld refinement pattern of undoped, Ce/Ni, Nd/Ni co-doped BaFe_12_O_19_ nanocompositions; (d) the 3d representation of co-doped samples.

The Rietveld refinement results (Fig. 2) further support this interpretation. The obtained goodness of fit factor (*χ*^2^) values are depicted in Table 1, showing a satisfactory agreement between the observed and calculated diffraction patterns and support the reliability of the extracted structural parameters. The lattice parameter (*a*) decreases from 5.9053 Å for BF to 5.8972 Å for both CNBF and NNBF, while the lattice parameter (*c*) decreases from 23.2648 Å for BF to 23.2280 Å for CNBF and 23.1912 Å for NNBF. Accordingly, the unit-cell volume decreases from 702.6095 Å^3^ for BF to 699.5678 Å^3^ for CNBF and 698.4603 Å^3^ for NNBF, clearly indicating a net contraction of the hexaferrite lattice upon co-doping. This contraction can be associated with the joint influence of ionic radius mismatch, cation redistribution, and charge-compensation processes arising from substitution of Ni^2+^ at Fe sites and Ce^4+^ or Nd^3+^ at Ba sites. The structural evolution revealed by XRD is important from both crystallographical and physical aspects. This is because the magnetoplumbite lattice is highly sensitive to local cation environment and axial distortion. The observed lattice parameters decrease, especially the stronger *c*-axis reduction in NNBF, suggests establishment of anisotropic chemical pressure induced by incorporation of the dopants into the hexaferrite blocks. In M-type BaFe_12_O_19_, the *c*-axis is closely related to the uniaxial anisotropy of the material, so that small distortions of the axis can influence the crystal-field surroundings and the exchange pathways among the Fe-containing sublattices. In this context the structural contraction is not only a geometric effect but probably also an early indicator of the changes that were observed later in the optical disorder, dielectric polarisation and magnetic reversal properties. NNBF exhibits a more significant lattice perturbation than CNBF, which is consistent with its larger Urbach energy and lower coercivity.

**Table 1:** Structural parameters of undoped, Ce/Ni co-doped, and Nd/Ni co-doped BaFe_12_O_19_ nanocompositions, including lattice constants (*a*), (*c*), unit-cell volume (*V*), crystallite size, internal parameter (*u*), bond length (*L*), and (*c*/*a*) ratio

Sample	*a* (Å)	*c* (Å)	*V* (Å^3^)	*c*/*a*	*u*	*L* (Å)	*χ* ^2^	Crystallite size (nm)
BF	5.9053	23.2648	702.6095	3.9397	0.27148	6.3159	1.3	58.08
CNBF	5.8972	23.2280	699.5678	3.9388	0.27149	6.3061	1.1	61.69
NNBF	5.8972	23.1912	698.4603	3.9326	0.27155	6.2977	1.2	55.84

The axial ratio (*c*/*a*) shows decreases slightly from 3.9397 for BF to 3.9388 for CNBF and 3.9326 for NNBF, indicating that the contraction is not perfectly isotropic and is more pronounced along the (*c*)-axis, especially for the Ni/Nd co-doped sample. Furthermore, the structural parameters (*u*) and (*L*), calculated using: *u* = (*a*^2^/3*c*^2^) + 0.25 and 
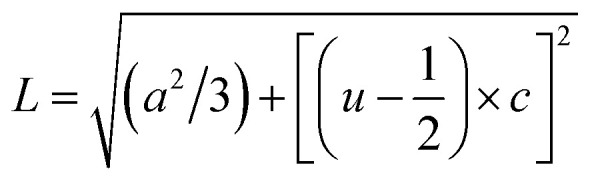
;^35,36^ support this interpretation. The (*u*) values show only a marginal increase from 0.27148 for BF to 0.27149 for CNBF and 0.27155 for NNBF, suggesting that the oxygen sublattice remains nearly unchanged and that the magnetoplumbite framework is structurally stable after co-doping. In contrast, the (*L*) parameter decreases from 6.3159 for BF to 6.3061 for CNBF and 6.2977 for NNBF, confirming a gradual contraction of the local structural geometry. The crystallite size calculated by the Scherrer formulae^37,38^ shows moderate variation among the samples, with values of 58.08 nm for BF, 61.69 nm for CNBF, and 55.84 nm for NNBF. The slightly larger crystallite size in CNBF suggests somewhat improved crystallite growth in the presence of Ce/Ni co-doping, whereas the smaller crystallite size of NNBF indicates that Nd/Ni co-doping may impose stronger inhibition on grain growth or introduce greater microstructural distortion.

**Fig. 3:**
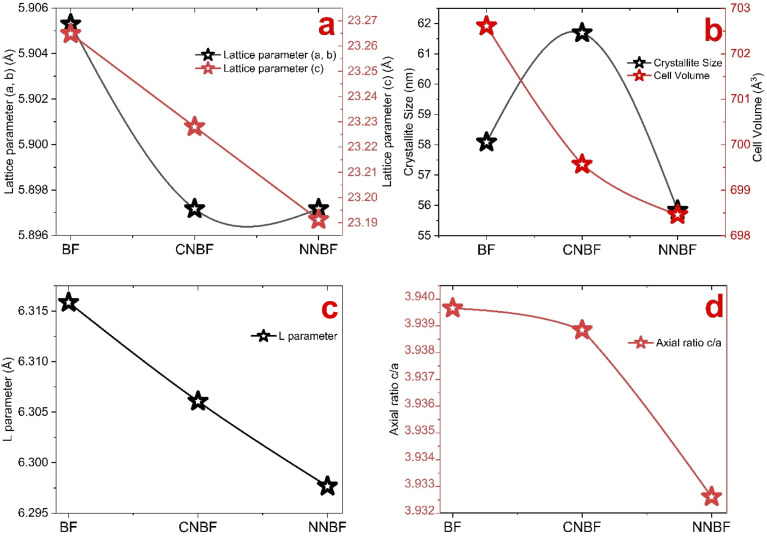
The impact of Ce/Ni and Nd/Ni co-doping on the structural parameters of BaFe_12_O_19_ nanocompositions: (a) lattice parameters *a* and *c*, (b) crystallite size and unit-cell volume, (c) lattice parameter *L*, and (d) axial ratio *c*/*a*.

The combined evolution of (*a*), (*c*), (*V*), (*c*/*a*), (*u*), (*L*), and crystallite size (Fig. 3 and Table 1), demonstrates that co-doping modifies the BaFe_12_O_19_ lattice at the atomic and microstructural levels without disrupting the fundamental M-type framework, with the contraction effect being more pronounced in the Ni/Nd co-doped sample.

### 3.2 Scanning electron microscopy analysis

**Fig. 4:**
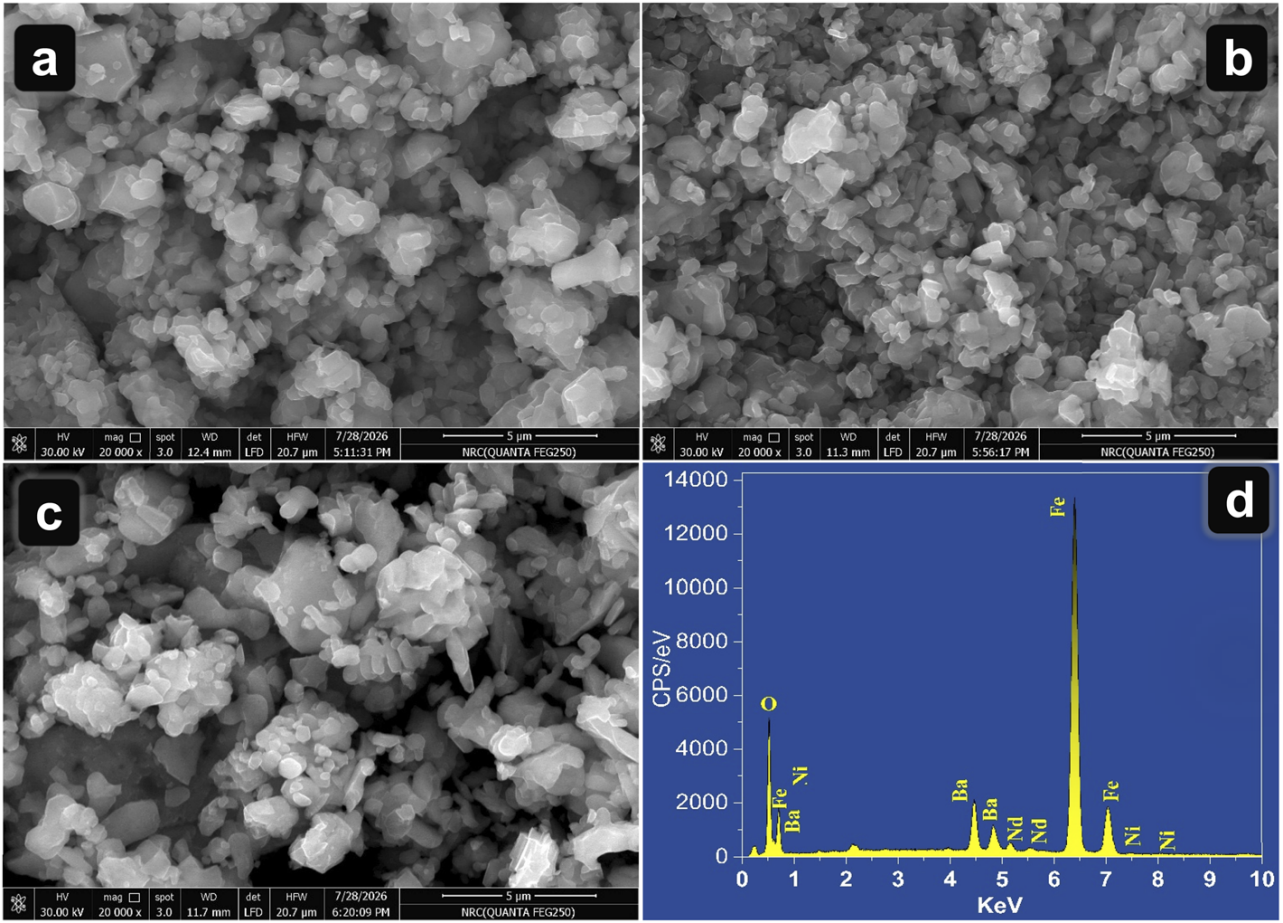
FESEM micrographs of (a) undoped; (b) Ce/Ni and (c) Nd/Ni co-doped BaFe_12_O_19_ nanocompositions respectively; (d) EDX spectra of Nd/Ni co-doped BaFe_12_O_19_ sample confirming elemental composition of the Nd/Ni co-doped sample.

The surface morphology of the pristine and co-doped BaFe_12_O_19_ samples was investigated using field-emission scanning electron microscopy (FESEM), and the representative micrographs are presented in Fig. 4. Additional high-resolution FESEM images, elemental mapping, EDX spectra, and particle size distribution histograms for all samples are provided in the SI (Fig. S1–S6). The acquired micrographs reveal that all compositions consist of agglomerated particles with irregular polyhedral morphology, which is characteristic of M-type hexaferrites synthesized by the sol–gel route.^39,40^ The pronounced agglomeration could be attributed to the high surface energy and strong magnetic dipole–dipole interactions among the ferrite particles.^41^ The pristine BaFe_12_O_19_ (BF) sample exhibits densely packed agglomerates composed of relatively coarse particles with an average particle diameter of approximately 0.77 µm, as determined from the particle size distribution analysis (Fig. S2). Upon Ce/Ni co-substitution (CNBF), the morphology becomes more homogeneous with finer particle assemblies and a narrower particle size distribution, yielding an average particle size of approximately 0.42 µm (Fig. S2). This behavior suggests that Ce/Ni co-doping suppresses excessive particle coarsening during calcination and promotes a more uniform microstructure. In contrast, the Nd/Ni co-doped sample (NNBF) exhibits a more heterogeneous morphology. Although the dominant particle population has an average diameter of approximately 0.48 µm (Fig. S2), the FESEM images reveal the coexistence of fine particles with a limited number of coarse particles measuring approximately 1.75–2.10 µm. In addition, several elongated particles are observed within the agglomerated structure, indicating a broader particle size distribution and a more heterogeneous growth behavior compared with the CNBF sample. These coarse particles were excluded from the statistical particle size distribution because they represent isolated grains rather than the predominant particle population and would disproportionately influence the calculated average particle size. The particle size distribution analysis demonstrates that the rare earth/Ni co-doping has a significant effect on the microstructure evolution of BaFe_12_O_19_. CNBF has the smallest average particle size and more uniform particle size distribution compared to the other investigated compositions; NNBF is morphologically more heterogeneous. These variations indicate that Ce and Nd modify the nucleation and growth mechanisms differently during synthesis, leading to different microstructural properties that are likely to affect the optical, dielectric and magnetic responses thereafter.

**Fig. 5:**
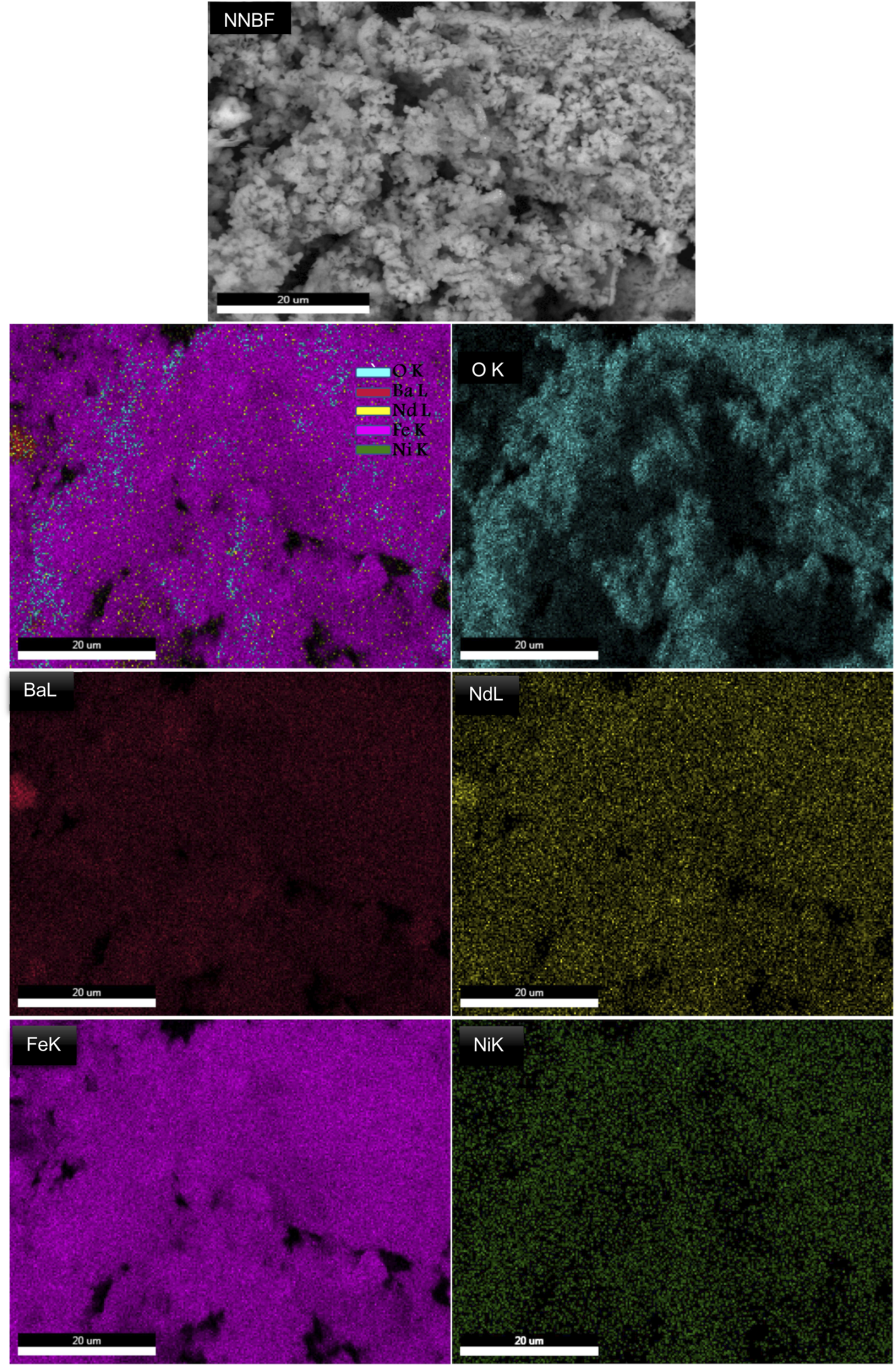
Elemental mapping images of NNBF sample showing the spatial distribution of Ba, Fe, O, Nd, and Ni throughout the sample.

The elemental composition of synthesized samples was further investigated by EDX spectroscopy. The EDX spectrum of the NNBF sample is shown in Fig. 4d; while the spectra of BF and CNBF are reported in the SI (Fig. S3a and b). The spectra confirmed the presence of the expected constituent elements with no detectable extraneous impurities. As expected, only Ba, Fe and O are detected in the BF sample while in the co-doped samples characteristic peaks corresponding to Ce and Ni (CNBF) and Nd and Ni (NNBF) are also observed confirming the successful incorporation of the dopant elements in the BaFe_12_O_19_ matrix. EDX analysis of NNBF samples reveals an at% distribution of 53.55 for oxygen, 42.01% for Fe and 3.74% for Ba, which is consistent with the nominal composition. The low atomic percentages of Nd and Ni successfully confirm the successful incorporation of the dopants in the hexaferrite lattice without any unassigned elemental impurities (Fig. 4d). The corresponding elemental mapping images (Fig. 5 and S4–S6) further demonstrate a homogeneous spatial distribution of Ba, Fe, O, and the respective dopant elements throughout the particles, indicating that the rare-earth and Ni ions are uniformly dispersed within the BaFe_12_O_19_ matrix. Thus, the FESEM analysis also shows that the dopant species change the crystal lattice and also affect the particle growth behavior during calcination. Especially, Ce/Ni co-doping results in a more uniform distribution of particles with the smallest average particle size, while Nd/Ni co-doping gives rise to a broader distribution of particles with isolated coarse and elongated particles, indicating different microstructural evolution driven by the different rare-earth ions.

### 3.3 FTIR spectra

**Fig. 6:**
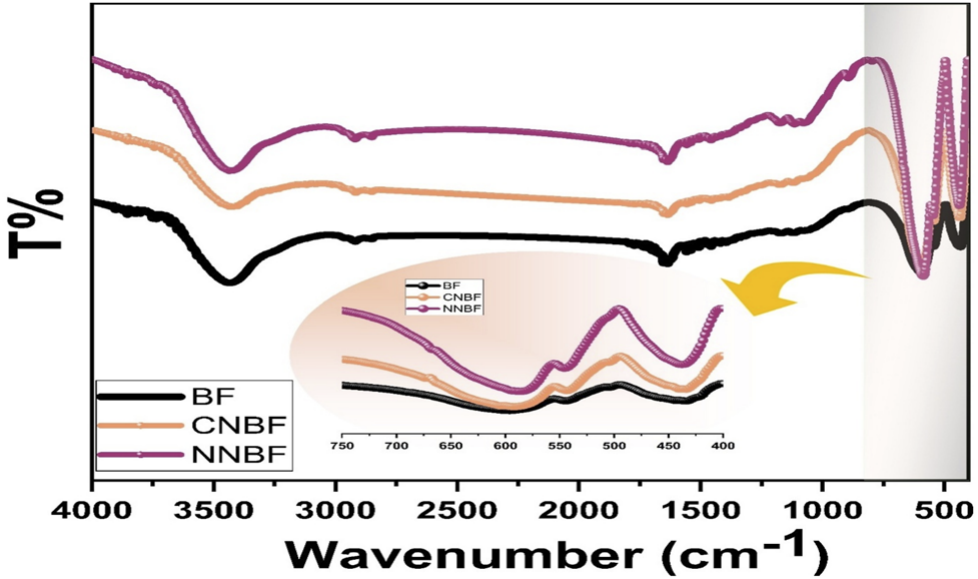
IR spectra of undoped and Ce/Ni, Nd/Ni co-doped BaFe_12_O_19_ nanocompositions; (inset) zoomed for the 400–900 cm^−1^ region.

The FTIR of BaFe_12_O_19_ (BF) and co-doped samples, CNBF Ba_0.97_Ce_0.03_Fe_11.97_Ni_0.03_O_19_ and Ba_0.97_Nd_0.03_Fe_11.97_Ni_0.03_O_19_ (NNBF), exhibit the characteristic vibrational bands of M-type barium hexaferrite together with absorption bands associated with residual hydroxyl and adsorbed species (Fig. 6). The main absorption bands are observed at approximately 434–437, 543–545, 586–595, 894, 1634–1636, 2842–2843, 2916–2918, and 3435–3436 cm^−1^. The presence of these bands confirms the formation of the ferrite framework and indicates that co-doping does not alter the fundamental vibrational features of the magnetoplumbite structure. The bands observed in the low-wavenumber region below about 650 cm^−1^ are attributed mainly to metal–oxygen vibrations within the FeO_6_ octahedral and FeO_4_ tetrahedral units of the hexaferrite lattice. In particular, the band near 434–437 cm^−1^ is associated with Fe–O vibrations at octahedral sites, whereas the bands near 543–545 cm^−1^ and 586–595 cm^−1^ are related to iron–oxygen polyhedra stretching vibrations in tetrahedral and octahedral coordination sublattices.^42–44^ These bands are characteristic of BaFe_12_O_19_ and confirm the preservation of the ferrite lattice after co-doping. The slight shifts in their positions from BF to CNBF and NNBF indicate that the substitution of Ni together with Ce or Nd modifies the local Fe–O bond environment. Such changes may arise from cation redistribution, lattice distortion, and variations in bond strength caused by the incorporation of dopant ions into the magnetoplumbite structure. The small shifts in the low-frequency bands also suggest that the local coordination geometry around Fe ions is altered without changing the overall crystal symmetry. For example, the band at 595 cm^−1^ in BF shifts to 592 cm^−1^ in CNBF and 586 cm^−1^ in NNBF, indicating a gradual modification of the metal–oxygen bond force constant, with the larger shift in NNBF implying a somewhat stronger perturbation of the local structure. This agrees well with the structural analysis, which showed that the NNBF sample undergoes the largest reduction in the (*c*-parameter) and unit-cell volume. The slightly greater shift observed for NNBF therefore supports the conclusion that Nd/Ni co-doping introduces stronger local distortion than Ce/Ni co-doping. The absorption band at 894 cm^−1^ appears at the same position in all samples, suggesting that this vibrational mode is not significantly affected by the dopant ions. The band near 1634–1636 cm^−1^ is ascribed to the bending vibration of H_2_O_ads_, while the broad band centered around 3435–3436 cm^−1^ is related to *ν*_s_ O–H of surface hydroxyl groups.^45,46^ In addition, weak bands are detected at 2842–2843 cm^−1^ and 2916–2918 cm^−1^ ascribed to *ν*_as_ and *ν*_s_ C–H, which may arise from trace residual organic species associated with the sol–gel synthesis route. The FTIR results confirm the formation of M-type BaFe_12_O_19_ in both undoped and co-doped samples and show that Ni/Ce and Ni/Nd co-doping induces only minor shifts in the Fe–O vibrational bands, reflecting local changes in the bonding environment that are in agreement with the XRD results, with NNBF displaying the most pronounced low-wavenumber shift and therefore a somewhat stronger local lattice perturbation than CNBF. The small shifts observed in FTIR spectra upon co-doping, are physically meaningful as they correspond to subtle changes to the Fe–O coordination environment. This dictates both superexchange interactions and the electronic structure near the band edges. In M-type hexaferrites, even small perturbations of Fe–O bond geometry can affect the magnetic exchange strength and the localized electronic states. The larger low-wavenumber shift obtained for NNBF thus corroborates the view that Nd/Ni co-doping perturbs the local lattice more strongly than Ce/Ni co-doping.

### 3.4 Optical properties

**Fig. 7:**
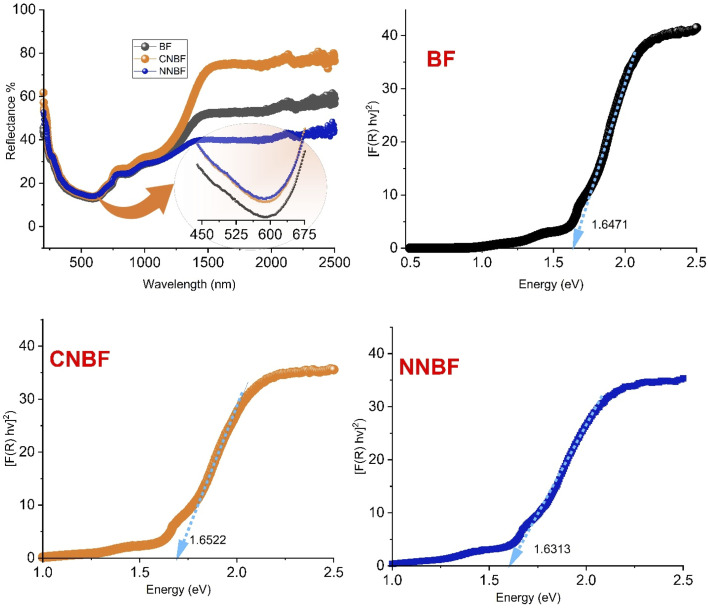
Diffuse reflectance spectra and corresponding Kubelka–Munk plot with extracted optical band gap energy values.

The diffuse reflectance (DR) spectra of the undoped BaFe_12_O_19_ (BF) and the co-doped samples, Ba_0.97_Nd_0.03_Fe_11.97_Ni_0.03_O_19_ (NNBF) and Ba_0.97_Ce_0.03_Fe_11.97_Ni_0.03_O_19_ (CNBF), measured in the wavelength range of approximately 200–2500 nm (Fig. 7a), show that co-doping significantly affects the optical response of M-type barium hexaferrite. All samples exhibit a similar overall spectral shape, with a modest reflectivity within the visible domain, succeeded by a monotonic rise toward the near-infrared region. Such behavior is characteristic of ferrite materials and reflects strong light absorption associated with electronic transitions involving Fe–O polyhedra in the magnetoplumbite lattice. The broad reflectance minimum observed in the visible region, particularly between about 450 and 675 nm, indicates substantial absorption arising mainly from charge-transfer O^2−^ → Fe^3+^ transitions, together with crystal-field-related electronic excitations of Fe^3+^ ions.^47,48^ A closer inspection of the spectra (inset) reveals that co-doping modifies both the depth and shape of the visible absorption band as well as the reflectance level in the near-infrared region. The BF sample exhibits the deepest reflectance minimum in the visible range, indicating relatively strong visible-light absorption. Both CNBF and NNBF samples show a comparable but slightly shallower minimum, whereas they present less pronounced absorption feature. At higher wavelengths, the differences become more distinct: CNBF displays the highest reflectance and a sharp rise above about 1300–1400 nm, reaching nearly 75–80% at long wavelengths, while BF shows intermediate reflectance values and NNBF remains the least reflective, with values around 40–45% in much of the near-infrared region. These variations indicate that Ce/Ni and Nd/Ni co-doping influence not only the intrinsic absorption processes but also the scattering behavior of the powders. The higher reflectance of CNBF may be related to its improved crystallinity, reduced Fe_2_O_3_ impurity contribution, and slightly larger crystallite size, as inferred from XRD and Rietveld refinement. In contrast, the relatively lower reflectance of NNBF may be associated with stronger lattice distortion, smaller crystallite size, and greater defect-related absorption.

For estimation of the optical band gap (*E*_g_), Kubelka–Munk function was applied: *F*(*R*_∞_) = (1 − *R*)^2^/2*R*.^49–52^ The *E*_g_ was then inferred from the Tauc relation: (*F*(*R*)*hν*)^*n*^ = *A*(*hν* − *E*_g_); (Fig. 7). Where (*hν*) is the photon energy, (*A*) is a proportionality constant, and (*n*) depends on the nature of the optical transition. A direct allowed transition (*n* = 2) was assumed for the band-gap estimation.^53,54^ The linear portion of the plot was fitted and extrapolated to the energy axis to estimate (*E*_g_). The extracted *E*_g_ values are 1.6471 eV for BF, 1.6522 eV for CNBF, and 1.6313 eV for NNBF. The *E*_g_ values show that co-doping leads to measurable changes in the band gap, indicating that the fundamental electronic structure of BaFe_12_O_19_ is preserved while the band-edge states are subtly modified. The small increase in band gap for CNBF relative to BF suggests a slight widening of the optical transition energy, which may be associated with improved phase purity, reduced defect concentration, and better crystallinity. Since structural disorder and secondary phases can introduce localized states near the band edges, their reduction tends to suppress sub-band-gap absorption and shift the absorption edge toward higher energy. XRD results showed that the Fe_2_O_3_ impurity is reduced in CNBF and that this sample possesses the largest crystallite size among the investigated compositions. By contrast, the NNBF sample exhibits the lowest band gap value, indicating a slight narrowing of the optical gap after Nd/Ni co-doping. This behavior may be attributed to stronger lattice distortion, greater local strain, and a comparatively higher concentration of defect-related states that can add mid-gap states in close proximity to the band edges. The Rietveld refinement showed that NNBF experiences the largest contraction of the (*c*)-parameter, the lowest unit-cell volume, and the lowest (*c*/*a*) ratio, suggesting that Nd/Ni co-doping perturbs the local structure more strongly than Ce/Ni co-doping. Such perturbation can modify Fe–O bond lengths, crystal-field splitting, and orbital hybridization, thereby slightly reducing the energy required for optical excitation. In addition, dopant-induced cation redistribution among the Fe sublattices may influence the overlap between Fe 3d and O 2p states, contributing further to the narrowing *E*_g_ value. These changes in (*E*_g_) indicate that rare-earth/Ni co-doping can tune the optical absorption edge of BaFe_12_O_19_ without altering its fundamental magnetoplumbite framework.

**Fig. 8:**
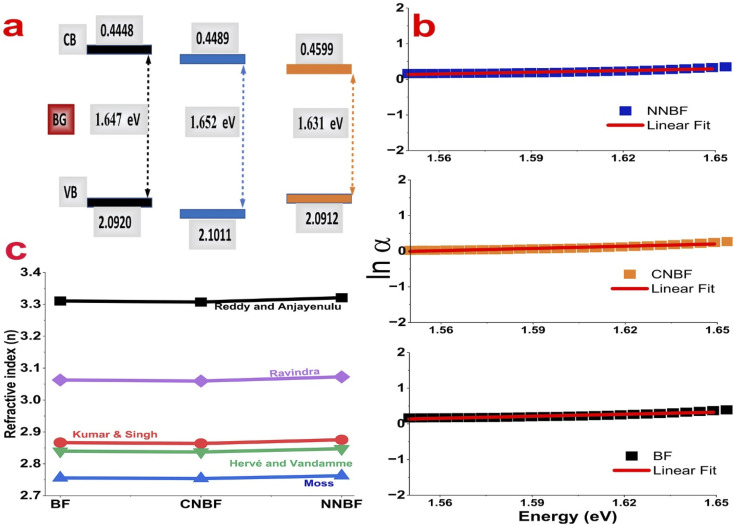
(a) CB and VB edges, (b) the Urbach energy plots and (c) refractive index values of undoped, and Ce/Ni, Nd/Ni co-doped BaFe_12_O_19_ nanocompositions.

To obtain further insight into the effect of Ce/Ni and Nd/Ni co-doping on the electronic structure, the valence-band *E*_VB_ and conduction-band *E*_CB_ edge positions were estimated using the electronegativity-based relations: *E*_VB_ = *X* − *E*_e_ + 0.5*E*_g_ and *E*_CB_ = *E*_VB_ − *E*_g_;^55,56^ where (*X*) is the absolute electronegativity of the semiconductor, *E*_e_ is the energy of free electrons on the hydrogen scale 4.5 eV, and *E*_g_ is the optical band gap. The calculated *E*_VB_ values are 2.0920 eV, 2.1011 eV, and 2.0912 eV for BF, CNBF, and NNBF, respectively, while the corresponding *E*_CB_ values are 0.4448 eV, 0.4489 eV, and 0.4599 eV (Fig. 8a). These results indicate that co-doping causes only slight shifts in the band-edge positions, confirming that the fundamental semiconducting nature of BaFe_12_O_19_ is preserved. Nevertheless, the observed changes reflect subtle modifications in the distribution of electronic states near the band edges induced by lattice distortion, cation redistribution, and defect chemistry. The slightly higher valence-band position and widened band gap of CNBF are consistent with its lower disorder and improved optical order, whereas the upward shift of the CB edge in NNBF, together with its smaller *E*_g_ and higher Urbach energy, suggests stronger perturbation of the electronic structure and a greater contribution from localized defect states. These findings further support the conclusion that Ni/Ce and Ni/Nd co-doping enables fine tuning of the optical and electronic properties of M-type BaFe_12_O_19_. To further examine the degree of structural disorder, the Urbach energy (*E*_U_) of the BF, CNBF, and NNBF samples was evaluated from the optical absorption edge (Fig. 8b). In disordered and polycrystalline materials, the absorption coefficient near the band edge commonly follows the ln *α*_*hν*_ = ln *α*_0_ + (*hν*/*E*_U_); where (*α*) is the absorption coefficient, (*hν*) is the photon energy, and (*E*_U_) represents the width of the band-tail states. The obtained (*E*_U_) values are 0.5266 eV for BF and 0.4768 eV for CNBF, and (0.6292) eV for NNBF. The lower Urbach energy of CNBF indicates a narrower tail of localized states and therefore a reduced structural and electronic disorder, which is well-reconciled with its improved crystallinity and larger crystallite size. In contrast, NNBF shows the highest Urbach energy, suggesting stronger disorder, higher defect density, and greater band-edge broadening. This behavior agrees with its more pronounced lattice contraction and smaller crystallite size, which indicate stronger structural distortion. The BF sample exhibits an intermediate value, reflecting a moderate degree of disorder in the undoped ferrite. A clear inverse relationship is observed between (*E*_g_) and (*E*_U_); CNBF shows the highest band gap and the lowest Urbach energy, whereas NNBF exhibits the lowest band gap and the highest Urbach energy. This confirms that the optical absorption edge is strongly influenced by disorder-induced localized states. The Urbach energy analysis indicates that Ce/Ni co-doping improves the optical order of BaFe_12_O_19_, while Nd/Ni co-doping increases structural and electronic disorder.

The band-gap and Urbach-energy results together indicate that the optical response is less dominated by a major reconstruction of the electronic structure and more by changes in band-edge disorder. The slight increase in *E*_g_ and the lower *E*_U_ for CNBF are indicative of a cleaner band edge with fewer localized tail states, consistent with enhanced structural coherence and reduced defect-related absorption. On the contrary, the reduced *E*_g_ and enhanced *E*_U_ of NNBF indicate that the Nd/Ni co-doping induces a broadened distribution of localized states, probably due to the stronger lattice distortion and larger defect perturbation. In this sense, the optical measurements give an independent probe of the same disorder trend as inferred from XRD and SEM, namely that Ce/Ni co-doping stabilizes a more ordered host matrix, whereas Nd/Ni co-doping.

The refractive index (*n*) of BF, CNBF, and NNBF was estimated from the optical band-gap values using several empirical models (Fig. 8c), including those of Reddy and Anjaneyulu, Kumar and Singh, Moss, Hervé and Vandamme, and Ravindra models.^31,57^ All models show a similar trend, with CNBF exhibiting the lowest refractive index and NNBF the highest. For example, the refractive index estimated using the Reddy and Anjaneyulu model changes from 3.31055 (BF) to 3.30726 (CNBF) and 3.32083 (NNBF), while the Moss relation gives 2.75582, 2.75369, and 2.76247, respectively. This behavior is inversely related to the optical band gap, where the slightly reduced *E*_g_ of NNBF leads to a higher refractive index, whereas the slightly widened band gap of CNBF produces a lower refractive index. These findings are consistent with the Urbach-energy results and further confirm that Ni/Nd and Ni/Ce co-doping provide a subtle means of tuning the optical polarizability and refractive response of BaFe_12_O_19_.

## 4 Dielectric properties and Ce/Ni, Nb/Ni doping effects

**Fig. 9:**
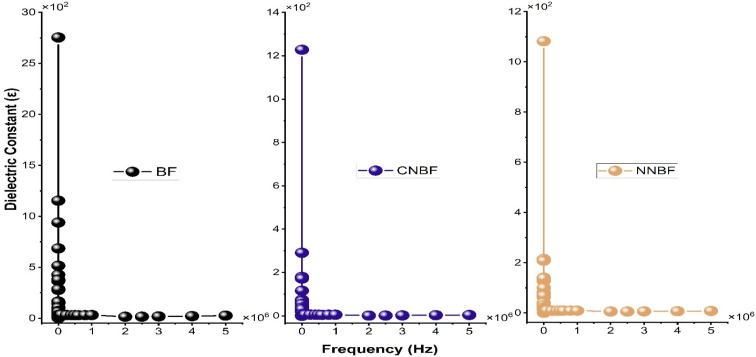
Frequency-driven variations in dielectric constant of undoped, Ce/Ni, Nd/Ni co-doped BaFe_12_O_19_ nanocompositions.

The dielectric behavior of ferrites can be analyzed through the frequency dependence of the dielectric constant (*ε*′), dielectric loss, (*ε*″), the loss tangent (tan *δ* = *ε*″/*ε*′), and the AC conductivity (*σ*). These parameters are closely related to polarization mechanisms, charge carrier hopping, and the microstructural characteristics of the material. The dielectric constant *ε*′ for BF, CNBF, and NNBF shows the typical dispersion behavior of ferrite materials. In all samples, *ε*′ is maximized in the low-frequency field and decline sharply upon increasing frequency, then tends to level off in the high-frequency area. Fig. 9 shows that at low frequencies the dielectric constant is highest for BF, reaching about 2750, while CNBF and NNBF show lower values of approximately 1230 and 1080, respectively. With increasing frequency, the dielectric constant drops rapidly for all compositions and reaches much lower values at high frequency, becoming nearly frequency-independent. At around (5 × 10^6^ Hz), the dielectric constant decreases to only a few units for all samples. This dielectric response is well-elucidated by the Maxwell–Wagner interfacial polarization model in conjunction with Koop's phenomenological theory. Within this framework, the ferrite is modeled as a heterogeneous system comprising semiconducting grains isolated by high-impedance grain boundaries. In the low-frequency regime, the migration and subsequent entrapment of charge carriers at these interfaces induce a pronounced space-charge polarization, thereby yielding elevated permittivity values. As the frequency increases, the carriers fail to synchronize with the rapidly oscillating external field, leading to a marked attenuation of the interfacial contribution. A comparison of the three samples indicates that co-doping decreases the dielectric constant, with the reduction being more pronounced for NNBF. This suggests that the introduction of Ni together with Ce or Nd modifies the polarization mechanism, possibly through changes in grain-boundary resistance, defect distribution, and electron-hopping probability between Fe^2+^/Fe^3+^ ions. The lower dielectric constant of the co-doped samples implies reduced space-charge accumulation and weaker interfacial polarization compared with the undoped BF sample.

**Fig. 10:**
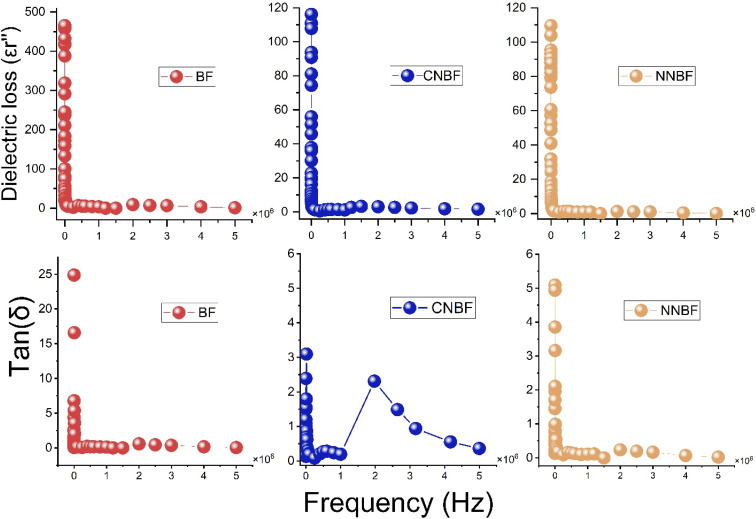
Frequency-driven variations in dielectric loss of undoped, Ce/Ni, Nd/Ni co-doped BaFe_12_O_19_ nanocompositions.

The (*ε*″) and (tan *δ*) represent energy dissipation within the material (Fig. 10). It showed that BF recorded the maximum *ε*″ values at low frequencies (∼450–500), CNBF shows moderate values (110–120), and NNBF is slightly lower (110). These high values at low frequencies indicate significant conduction and space-charge losses. As frequency increases, *ε*″ sharply decreases, approaching near zero at high frequencies, reflecting diminished energy dissipation when charge carriers cannot follow the alternating field. The tan *δ* = *ε*″/*ε*′ values follow a similar trend and are highest for BF at low frequencies (around 25), indicating substantial energy loss per cycle, whereas CNBF and NNBF show lower values (∼3–5). At higher frequencies (≥10^5^ Hz), tan *δ* becomes very small for all samples, signifying minimal energy loss. The lower dielectric loss in CNBF and NNBF suggests that co-doping reduces conduction losses and space-charge polarization. One reason is the internal charge compensation effect, where Ni^2+^ substitution at Fe^3+^ sites introduce acceptor states, while Ce^4+^/Nd^3+^ substitution at Ba^2+^ sites introduce donor-type defects. This can be expressed using Kröger–Vink notation: 
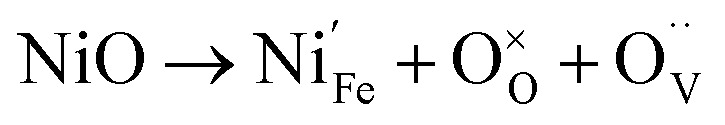
; 
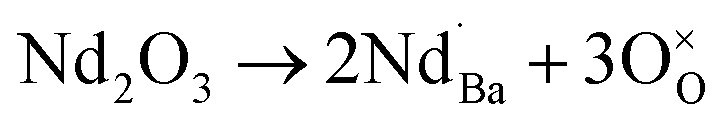
; 
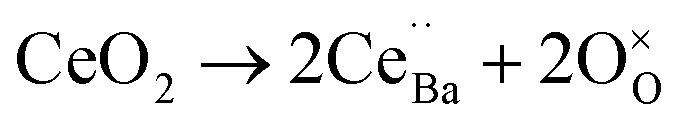
. These reactions indicate that doping introduces defects such as 
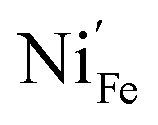
, 
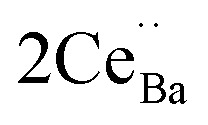
 and oxygen vacancies 
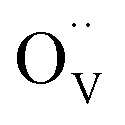
; which influence charge carrier movement. Effective internal compensation reduces free charge carriers available for hopping, thus decreasing conduction losses and dielectric loss. The dielectric behavior of the co-doped samples reveals that the primary effect of Ni/rare-earth substitution is to suppress the extrinsic interfacial polarization, rather than to enhance the intrinsic dipolar polarization. In ferrites, the high low-frequency permittivity is generally ascribed to the accumulation of charge carriers at the poorly conducting grain boundaries. The decrease of *ε*′, *ε*″ and tan *δ* after co-doping thus indicates that the dopants modify the defect landscape and Fe^2+^/Fe^3+^ hopping pathways limiting long-range space-charge build-up. The enhanced suppression of NNBF is consistent with a more defect-perturbed and structurally distorted matrix, whereas the relatively balanced response of CNBF indicates that Ce/Ni co-doping reduces dielectric loss while not excessively disrupting structural coherence. This interpretation also agrees with the optical disorder analysis that reveals that CNBF has a lower Urbach energy than NNBF. A more detailed separation of grain and grain-boundary contributions through complex impedance or Cole–Cole analysis would be useful in future work to further clarify the microstructural origin of the dielectric response.

**Fig. 11:**
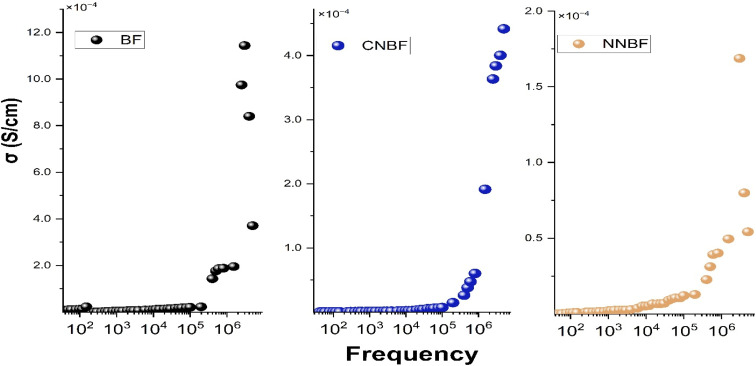
Frequency-driven variations in the conductivity for undoped, Ce/Ni, Nd/Ni co-doped BaFe_12_O_19_ nanocompositions.

AC conductivity (*σ*) is related to the dielectric parameters *via*: *σ*(*ω*) = *ω* × *ε*_0_ × *ε*″; where *ε*_0_ is the permittivity of free space and *ω* is the angular frequency (*ω* = 2π*f*). The *σ* plots for BF, CNBF, and NNBF (Fig. 11) all exhibit a progressive increment in *σ* with frequency. At around 10^2^ Hz, BF has a conductivity on the order of 10^−4^ S cm^−1^, increasing steadily to about 11.4 × 10^−4^ S cm^−1^ at higher frequencies. This frequency-dependent increase in *σ* can be described by the Jonscher power law: *σ*(*ω*) = *σ*_dc_ + *Aω*^*n*^; where *σ*_dc_ is the DC conductivity, *A* is a constant, and 0 < *n* < 1. CNBF and NNBF start with slightly lower conductivity values, around 1.89 × 10^−4^ S cm^−1^ and 1.25 × 10^−4^ S cm^−1^ respectively at low frequencies, and increase to about 4.41 × 10^−4^ and 1.68 × 10^−4^ S cm^−1^ at the highest frequencies measured. The increase of *σ* with frequency is typical of hopping conduction mechanisms in ferrites, where charge carriers hop between Fe^2+^ and Fe^3+^ ions. The lower *σ* at low frequencies in CNBF and NNBF compared to BF suggests that co-doping reduces the number of free charge carriers or their mobility, consistent with the observed reduction in dielectric loss.

### 4.1 Magnetic properties

**Fig. 12:**
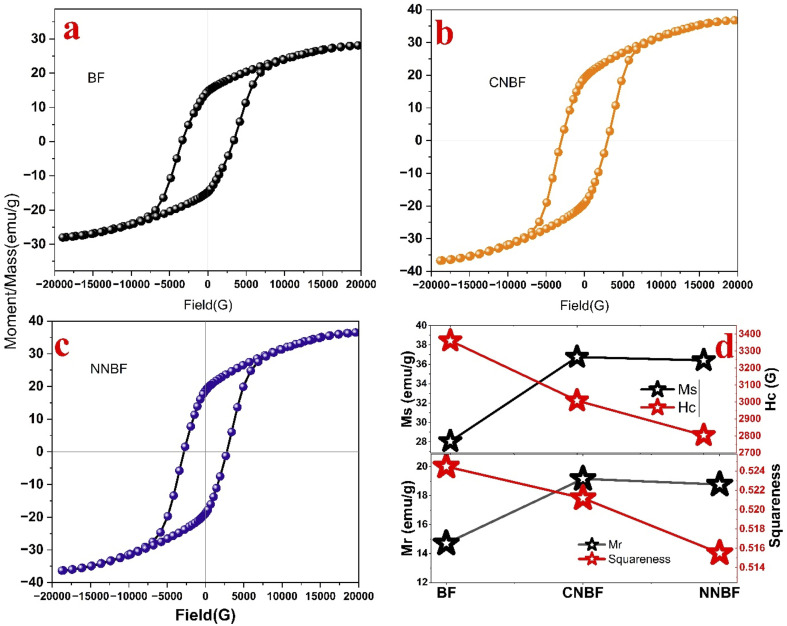
(a–c) Magnetic hysteresis loops (*M*–*H*), of undoped, Ce/Ni, Nd/Ni co-doped BaFe_12_O_19_ nanocompositions; (d) effect of co-doping on the *M*_s_, *H*_c_, *M*_r_ and *Q* of BaFe_12_O_19_.

The room-temperature magnetic hysteresis loops obtained from VSM measurements confirm the ferromagnetic behavior of all samples (Fig. 12). The undoped BaFe_12_O_19_ (BF) sample exhibits (*M*_s_ = 28.04) emu g^−1^; upon co-doping, the magnetic response is markedly modified. The Ce/Ni co-doped sample (CNBF) shows the highest saturation magnetization (36.75 emu g^−1^), followed closely by the Nd/Ni co-doped sample (NNBF, 36.41 emu g^−1^). *M*_s_ enhancement could be mainly linked with that dopant-induced cation rearrangement among the five nonequivalent magnetic sublattices of M-type BaFe_12_O_19_, namely 12k, 2a, 2b, 4f_1_, and 4f_2_. In the magnetoplumbite structure, the net magnetic moment arises from the difference between the spin-up sublattices 12k, 2a, and 2b, and the spin-down sublattices (4f_1_ and 4f_2_). Ni^2+^ ions are generally reported to preferentially occupy octahedral sites, especially 4f_2_ and/or 12k-related positions, depending on the synthesis conditions.^58^ If Ni^2+^ enters a spin-down site such as 4f_2_, the opposing sublattice moment is reduced, leading to an increase in the net magnetization. The rare-earth dopants may also affect the magnetic behavior through their intrinsic magnetic moments and through their influence on the local crystal chemistry. Nd^3+^ possesses a relatively large effective magnetic moment of about 3.62*µ*_B_, arising from its 4f^3^ electronic configuration, and therefore can contribute to the magnetic response indirectly through 4f–3d exchange interactions, lattice distortion, and modification of Fe^3+^ cation distribution. In contrast, cerium may exist as Ce^3+^ or Ce^4+^ depending on the local chemical environment. Ce^3+^ has an effective magnetic moment of about 2.54*µ*_B_, whereas Ce^4+^ is essentially nonmagnetic (4f^0^, *µ*_eff_ ≈ 0*µ*_B_). Therefore, the direct magnetic contribution of cerium is expected to be smaller than that of Nd^3+^, especially if Ce is predominantly present as Ce^4+^. The slightly higher *M*_s_ observed for CNBF compared with NNBF is thus more likely related to improved phase purity, reduced Fe_2_O_3_ impurity, and better crystallinity rather than to a larger intrinsic rare-earth magnetic moment. In addition, the more homogeneous microstructure, narrower particle-size distribution, and slightly larger crystallite size of the CNBF sample relative to BF and NNBF may collectively reduce surface spin disorder and strengthen magnetic exchange interactions, thus giving rise to its higher saturation magnetization.

In contrast, the coercivity decreases from 3360.9 Oe for BF to 3007.8 Oe for CNBF and 2803.5 Oe for NNBF may be discussed in terms of both intrinsic and extrinsic factors. In M-type hexaferrites, coercivity is strongly influenced by magnetocrystalline anisotropy, cation occupancy, crystallite size, strain, and defect-assisted domain-wall pinning. The Scherrer crystallite size changes from 58.08 nm for BF to 61.69 nm for CNBF and 55.84 nm for NNBF. The decrease in (*H*_c_) for CNBF may be partly associated with its larger crystallite size, since larger crystallites generally reduce the barrier for magnetization reversal and can facilitate easier domain-wall motion. However, the NNBF sample exhibits the lowest coercivity despite having the smallest crystallite size, indicating that crystallite size alone cannot explain the observed coercivity trend. This suggests that the reduction in (*H*_c_) is dominated not only by particle-size effects but also by dopant-induced changes in magnetocrystalline anisotropy. In particular, substitution of Fe^3+^ by Ni^2+^ at anisotropy-sensitive sites may weaken the magnetocrystalline anisotropy of the hexaferrite lattice, thereby lowering the coercive field. This effect appears to be stronger in NNBF, where the more pronounced lattice contraction and reduced (*c*/*a*) ratio suggest a greater structural perturbation and, consequently, a larger reduction in anisotropy or pinning strength. The presence of residual Fe_2_O_3_ may also contribute to the coercivity behavior. The XRD results indicate that the Fe_2_O_3_ contribution is reduced in CNBF but relatively higher in NNBF. This higher content, together with the stronger lattice distortion and more heterogeneous morphology of NNBF, likely contributes to its lower *H*_c_ by disturbing the magnetic homogeneity of the hard ferrite matrix and creates magnetically softer or weakly anisotropic regions, which may reduce the effectiveness of domain-wall pinning and facilitate reversal under an applied field. From a mechanistic standpoint, the magnetic performance enhancement after co-doping is governed by the interplay of intrinsic sublattice and extrinsic microstructural factors. Intrinsically, substitution of Ni at Fe sites can modify the balance between spin-up and spin-down sublattice moments, and a favorable redistribution can lead to an increase in net ferrimagnetic moment. Rare-earth doping at the Ba site, which is not directly involved in the Fe magnetic network, can have an indirect influence on the magnetization by perturbing local crystal fields, bond geometries and cation distributions. Extrinsically, the reduction of Fe_2_O_3_ secondary contribution, larger crystallite size and more homogenous morphology of CNBF are likely to reduce the spin disorder and improve the microstructural uniformity across neighboring grains. This gives a physically consistent explanation to the reason why CNBF has the highest saturation magnetization and maximum energy product. By contrast, the stronger lattice perturbation and greater morphological heterogeneity of NNBF likely reduce the uniformity of magnetic reversal and helps in explaining the larger decrease of its coercivity, despite its similarly *M*_s_ enhancement. The remanent magnetization follows the same trend as *M*_s_, increasing from 14.71 for BF to 19.16 emu g^−1^ for CNBF and 18.77 emu g^−1^ for NNBF, which reflects stronger residual zero-field magnetization. In contrast, the squareness ratio remains nearly unchanged, with values of 0.5245, 0.5212, and 0.5155 for BF, CNBF, and NNBF, respectively. These values are close to 0.5, which is consistent with randomly aligned uniaxial single-domain particles according to the Stoner–Wohlfarth model.^59,60^ The nearly constant squareness ratio suggests that co-doping modifies the magnitude of the magnetic parameters without fundamentally changing the overall magnetization reversal mode.

To further evaluate the anisotropy behavior, the high-field magnetization data were analyzed using the law of approach to saturation (LAS): 
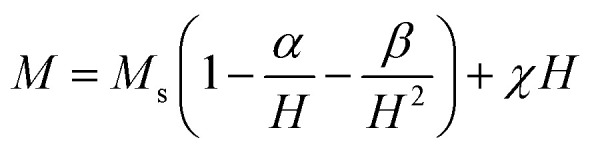
;^61,62^ where the *α*/*H* term is associated with microstructural inhomogeneity and domain-related effects, the *β*/*H*^2^ term corresponds to the magnetocrystalline anisotropy contribution, and *χH* represents the forced magnetization term. Since the high-field region (*H* > 15 kOe) showed a linear dependence of *M* on 1/*H*^2^, the 1/*H* term was neglected and the simplified relation: 
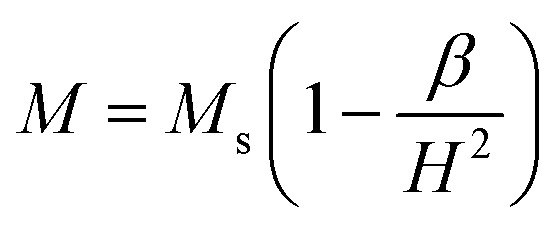
 was used. For a uniaxial hexagonal ferrite, *β* is related to the effective anisotropy constant (*K*_eff_) by: 
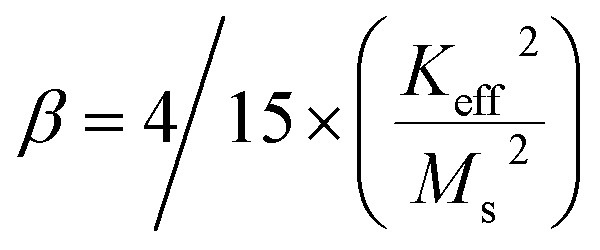
;^19,61^ and the anisotropy field (*H*_a_) is obtained from: *H*_a_ = 2*K*_eff_/*M*_s_.^19^ The calculated *K*_eff_ values increase from 1.444 × 10^6^ erg cm^−3^ for BF to 1.826 × 10^6^ erg cm^−3^ for CNBF and 1.828 × 10^6^ erg cm^−3^ for NNBF, showing that co-doping enhances the intrinsic anisotropy energy of the magnetoplumbite lattice. This increase can be attributed to Ni substitution at anisotropy-sensitive Fe sites together with rare-earth-induced lattice distortion and modified crystal-field effects around the Fe–O polyhedra. The slightly higher *K*_eff_ of NNBF is consistent with its stronger lattice contraction and lower *c*/*a* ratio, which suggest a more pronounced perturbation of the anisotropy-sensitive sublattices. Despite the increase in *K*_eff_, the anisotropy field (*H*_a_) decreases slightly after co-doping because *H*_a_ = 2*K*_eff_/*M*_s_;^19^ and the increase in *M*_s_ is proportionally stronger than the increase in *K*_eff_. This helps explain why the coercivity decreases even though the intrinsic anisotropy constant increases. In other words, the magnetic reversal process is controlled not only by intrinsic anisotropy but also by microstructural factors such as impurity content, strain, defect structure, and domain-wall pinning. This effect is especially evident in NNBF, which combines the highest *K*_eff_ with the lowest *H*_c_, indicating that its stronger structural distortion and higher Fe_2_O_3_ contribution reduce the effectiveness of pinning barriers during magnetization reversal. The maximum energy product BH_max_, obtained from the second quadrant of the hysteresis loop, also improves after co-doping. The measured values are 2.40 × 10^5^ erg g^−1^ for BF, 2.80 × 10^5^ erg g^−1^ for CNBF, and 2.67 × 10^5^ erg g^−1^ for NNBF. Therefore, BH_max_ increases by about 16.9% for CNBF and 11.3% for NNBF relative to the undoped sample. This enhancement is mainly associated with the simultaneous increase in *M*_s_ and *M*_r_, which improves the magnetic energy storage capability of the material. Among the investigated samples, CNBF exhibits the most favorable permanent-magnet characteristics, owing to its highest magnetization, lower Fe_2_O_3_ impurity contribution, and better crystallite growth. Although the coercivity decreases after co-doping, it remains sufficiently high to preserve hard magnetic behavior. These results demonstrate that Ni/Ce and Ni/Nd co-doping is a valid approach for tuning the magnetic characteristics of BaFe_12_O_19_ with the Ce/Ni co-doped sample showing the most balanced magnetic enhancement (Table 2). The room temperature magnetometry presented here shows the effect of co-doping on hard-magnetic behavior, but temperature dependent magnetic measurements, such as low temperature hysteresis loops, would be beneficial in future work to further determine the contributions of anisotropy evolution, thermal activation, and rare-earth based magnetic contributions.

**Table 2:** Magnetic parameters of room temperature of undoped, Ce/Ni and Nd/Ni co-doped BaFe_12_O_19_ nanocompositions

Sample	*M*_s_ (emu g^−1^)	*M*_r_ (emu g^−1^)	*H*_c_ (Oe)	*M*_r_/*M*_s_	*K*_eff_ (erg cm^−3^)	*H*_a_ (Oe)	BH_max_ (erg g^−1^)
BF	28.04	14.71	3360.9	0.5245	1.44 × 10^6^	18 545.32	2.40 × 10^5^
CNBF	36.75	19.16	3007.8	0.5212	1.826 × 10^6^	17 993.38	2.80 × 10^5^
NNBF	36.41	18.77	2803.5	0.5155	1.828 × 10^6^	17 963.71	2.67× 10^5^

A coherent physical picture emerges when the structural, optical, dielectric, and magnetic results are viewed together. Co-doping alters the BaFe_12_O_19_ lattice through coupled cation distribution, defect compensation, and local strain, evident in anisotropic lattice contraction, change in band gap and Urbach energy, suppression of low frequency interfacial polarization, and enhancement of *M*_s_ and BH_max_. Among the compositions studied, CNBF had the best combination of lowest secondary-phase contribution, slightly larger crystallite size, reduced disorder, more coherent morphology, lower dielectric loss, and the highest magnetic energy product, implying a more coherent ferrite matrix overall. On the other hand, NNBF has stronger local perturbation and disorder. Thus, the multifunctional behavior is more governed by how the co-doping improves the structural coherence, reduces disorder due to defects, and improved grain connectivity in the host lattice than by the nominal dopant concentration alone.

### 4.2 Mechanistic correlation of structure and multifunctional properties

The combined structural, optical, dielectric and magnetic results suggest that the multifunctional behavior of the Ce/Ni- and Nd/Ni-co-doped BaFe_12_O_19_ nanocompositions originates from a common dopant-induced structural perturbation rather than from independent modifications of the individual properties. Although co-doped compositions keep the magnetoplumbite structure, XRD and Rietveld refinement results show systematic lattice contraction and local structural distortion due to Ce/Ni and Nd/Ni substitution. Such changes, which modify the local Fe–O coordination environment as discussed in the structural analysis, are also further supported by the slight shifts in the FTIR metal–oxygen vibration bands. The structural modifications alter the Fe–O–Fe superexchange interactions, thereby influencing the optical, dielectric, and magnetic responses simultaneously. The optical measurements support this interpretation. The slightly higher optical band gap together with lower Urbach energy of the Ce/Ni co-doped sample show reduction in localized band-tail states and therefore less structural and electronic disorder. In contrast, the Nd/Ni co-doped sample shows the highest Urbach energy and the smallest optical band gap, indicating that a wider distribution of defect-related localized states is generated by stronger lattice disturbance. These optical results are consistent with the structural modifications revealed by XRD and the FESEM observations, where the Ce/Ni co-doped sample exhibits a more homogeneous particle distribution, while the Nd/Ni co-doped sample displays a broader particle-size distribution, isolated coarse and elongated particles which reflects a higher degree of microstructural heterogeneity. The dielectric behavior follows the same trend discussed previously, with reduced dielectric constant and dielectric loss indicating suppressed interfacial polarization and fewer mobile charge carriers.

Consistent with the magnetic analysis, the structural modifications induced by co-doping influence the ferrimagnetic exchange interactions through changes in magnetic sublattice occupation and local crystal-field symmetry. The higher *M*_s_ obtained for the CNBF sample is attributed to favorable cation redistribution, reduced structural disorder, and the more homogeneous microstructure with a narrower particle-size distribution, which collectively promote stronger magnetic exchange coupling between neighboring particles. In contrast, the broader particle-size distribution together with the existence of isolated coarse and elongated particles in the NNBF sample reflects greater microstructural heterogeneity, that, combined with the increased lattice distortion and defect states, contributes to the observed reduction in coercivity despite the saturation magnetization enhancement. Thus, the present results demonstrate that Ce/Ni and Nd/Ni co-substitution modifies BaFe_12_O_19_ through a coupled mechanism involving lattice distortion, defect compensation, cation redistribution, microstructural evolution, and modifications of the Fe–O–Fe super-exchange interactions. Rather than independently affecting the structural, optical, dielectric, or magnetic characteristics, these factors collectively determine the multifunctional response of the material. Among the investigated compositions, Ce/Ni co-doping exhibits the most advantageous structural stability, microstructure homogeneity, electronic disorder reduction, dielectric loss decrease, magnetic exchange interactions enhancement, and permanent-magnet performance improvement among the investigated compositions. More broadly, the significance of the present study extends beyond documenting property variations following co-doping. The results show that by comparing Ce/Ni and Nd/Ni co-substitution under the same synthesis conditions, the degree of lattice distortion, microstructural evolution, electronic disorder, dielectric polarization and magnetic exchange interactions are governed by the choice of rare-earth dopant. This comparative analysis establishes clearer structure–property relationships in M-type barium hexaferrite and provides useful guidance for selecting dopant combinations for targeted multifunctional applications.

## 5 Conclusion

Undoped BaFe_12_O_19_ (BF) and Ni/rare-earth co-doped BaFe_12_O_19_ nanocompositions, namely Ba_0.97_Ce_0.03_Fe_11.97_Ni_0.03_O_19_ and Ba_0.97_Nd_0.03_Fe_11.97_Ni_0.03_O_19_ were successfully synthesized *via* the sol–gel route and their structural, morphological, optical, dielectric, and magnetic properties were investigated. XRD and Rietveld refinement confirmed that all samples retained the hexagonal magnetoplumbite M-type structure, while co-doping induced measurable lattice contraction, with the structural distortion being more pronounced for the Nd/Ni co-doped sample. Optical studies revealed that Ce/Ni co-doping slightly widened the band gap and reduced electronic disorder, whereas Nd/Ni co-doping reduced the band gap and increased defect-related localized states, consistent with the XRD and FESEM observations. Dielectric measurements demonstrated that co-doping effectively suppressed low-frequency dielectric polarization and dielectric loss, which can be attributed to reduced space-charge polarization, modified grain-boundary behavior, and internal donor–acceptor charge compensation. Magnetic measurements demonstrated that the co-doping enhanced ferrimagnetic performance, with the Ce/Ni co-doped sample exhibiting the most favorable combination of saturation magnetization, coercivity, and maximum energy product. These results demonstrate that Ni/rare-earth co-doping is an effective strategy for tuning the multifunctional features of M-type BaFe_12_O_19_. Among the investigated compositions, Ce/Ni co-doping provides the most balanced enhancement by combining improved structural order, reduced dielectric loss, enhanced magnetic performance and a homogeneous microstructure. Mechanistically, the observed property evolution can be attributed to the combined effects of cation redistribution, lattice distortion, charge compensation, and microstructural evolution induced by Ni/rare-earth co-doping, which collectively modify the Fe–O–Fe superexchange interactions and govern the coupled multifunctional response. Overall, these findings reveal that the choice of rare-earth dopant governs the extent of lattice distortion, microstructural development, electronic disorder, dielectric polarization and magnetic exchange interactions in BaFe_12_O_19_. The systemic comparison of Ce/Ni and Nd/Ni co-substitution under the same synthesis conditions establishes clear structure–property relationships that contribute to a better understanding of multifunctional M-type barium hexaferrites and provide useful guidance for the design of substituted hexaferrites with tailored functional properties.

## Author contributions

Mohammed Wahba: conceptualization, methodology, validation, formal analysis, investigation, data curation, writing – original draft, review& editing.

## Conflicts of interest

The authors declare no competing interests.

## Supplementary Material

RA-OLF-D6RA03457H-s001

## Data Availability

The data supporting the findings of this study are available from the corresponding author upon reasonable request. Supplementary information (SI): additional FESEM images, particle size distributions, EDS spectra, and elemental mapping corresponding to the BF, CNBF, and NNBF samples, as well as the instruments used for the characterization. See DOI: https://doi.org/10.1039/d6ra03457h.

## References

[cit1] Matran W. M., Mustapha M., Nor M. F. (2024). The influences of additives in M− type (Ba and Sr) hexaferrites' microstructure, sintering and magnetic properties: A review. Mater. Today: Proc..

[cit2] Alsmadi A., Bsoul I., Mahmood S., Alnawashi G., Prokeš K., Siemensmeyer K., Klemke B., Nakotte H. (2013). Magnetic study of M-type doped barium hexaferrite nanocrystalline particles. J. Appl. Phys..

[cit3] Kumar S., Prakash J., Verma A., Jasrotia R. (2023). A comprehensive review of synthesis, properties, and applications of BaFe12O19 hexaferrites. Mater. Today: Proc..

[cit4] Chopra A., Arya N., Amir M., Bairagi S., Baykal A., Khan G. S., Ali S. W. (2024). Leveraging BaFe12O19/PANI nanocomposite incorporated cotton fabric as an effective EMI shielding flexible material for X-band frequency. ACS Appl. Electron. Mater..

[cit5] Pullar R. C. (2012). Hexagonal ferrites: a review of the synthesis, properties and applications of hexaferrite ceramics. Prog. Mater. Sci..

[cit6] Siegrist T., Vanderah T. A. (2003). Combining Magnets and Dielectrics: Crystal Chemistry in the BaO− Fe2O3− TiO2 System. Eur. J. Inorg. Chem..

[cit7] Atkare S. S., Alone S. T., Fulari A. V., Dhage V. N., Kadam R., Shirsath S. E., Mane M. L. (2025). Design of multifunctional rare earth Dy–Ce substituted BaFe12O19 nanoparticles for X-band microwave absorption and EMI shielding. Inorg. Chem. Commun..

[cit8] Ashiq M. N., Shakoor S., Najam-ul-Haq M., Warsi M. F., Ali I., Shakir I. (2015). Structural, electrical, dielectric and magnetic properties of Gd-Sn substituted Sr-hexaferrite synthesized by sol–gel combustion method. J. Magn. Magn. Mater..

[cit9] Buzinaro M., Costa B., Ivanov M., Cunha G., Macêdo M., Angélica R., Ferreira N. (2022). Investigations on the structural and magnetic properties of Ba1-xGdxFe12O19 (0≤ x≤ 0.15) nanoparticles. J. Magn. Magn. Mater..

[cit10] Trukhanov A., Kostishyn V., Panina L., Jabarov S., Korovushkin V., Trukhanov S., Trukhanova E. (2017). Magnetic properties and Mössbauer study of gallium doped M-type barium hexaferrites. Ceram. Int..

[cit11] Dhage V. N., Mane M., Keche A., Birajdar C., Jadhav K. (2011). Structural and magnetic behaviour of aluminium doped barium hexaferrite nanoparticles synthesized by solution combustion technique. Phys. B.

[cit12] Slimani Y., Baykal A., Manikandan A. (2018). Effect of Cr3+ substitution on AC susceptibility of Ba hexaferrite nanoparticles. J. Magn. Magn. Mater..

[cit13] Vinnik D., Kokovkin V., Volchek V., Zhivulin V., Abramov P., Cherkasova N., Sun Z., Sayyed M., Tishkevich D., Trukhanov A. (2021). Electrocatalytic activity of various hexagonal ferrites in OER process. Mater. Chem. Phys..

[cit14] Zhivulin V., Zaitseva O., Trofimov E., Zabeivorota N., Gavrilyak M., Podgornov F., Vinnik D., Almessiere M., Slimani Y., Baykal A. (2021). Anisotropy of the electrical properties of a single crystal of BaFe11. 25Ti0. 75O19 M-type barium hexaferrite. J. Solid State Chem..

[cit15] Wang L., Zhang J., Zhang Q., Xu N., Song J. (2015). XAFS and XPS studies on site occupation of Sm3+ ions in Sm doped M-type BaFe12O19. J. Magn. Magn. Mater..

[cit16] Mulay V., Sinha A. (1970). Synthesis and properties of some new ferrites of formula LA3+ ME2+ FE311+ O19. Indian J. Pure Appl. Phys..

[cit17] Rai G. M., Iqbal M., Kubra K. (2010). Effect of Ho3+ substitutions on the structural and magnetic properties of BaFe12O19 hexaferrites. J. Alloys Compd..

[cit18] Li C.-J., Huang B.-N., Wang J.-N. (2013). Effect of aluminum substitution on microstructure and magnetic properties of electrospun BaFe12O19 nanofibers. J. Mater. Sci..

[cit19] Manglam M. K., Shukla A., Mallick J., Yadav M. K., Kumari S., Zope M., Kar M. (2022). Enhancement of coercivity of M-type barium hexaferrite by Ho doping. Mater. Today: Proc..

[cit20] Kishor G., Bhowmik R., Kaushik S., Babu P. (2024). Investigation of structural phase stability, modified magnetic spin order and low temperature spin glass-like phase transitions in Sc-doped M-type BaFe12O19 hexaferrite. Phys. B.

[cit21] Alzaid M. (2021). Enhancement in Optical Properties of Lanthanum-Doped Manganese Barium Hexaferrites under Different Substitutions. Adv. Condens. Matter Phys..

[cit22] Todkar G., Kunale R., Kamble R., Batoo K. M., Ijaz M., Imran A., Hadi M., Raslan E., Shirsath S. E., Kadam R. (2021). Ce–Dy substituted barium hexaferrite nanoparticles with large coercivity for permanent magnet and microwave absorber application. J. Phys. D: Appl. Phys..

[cit23] Atif M., Husnain H. U., Rehman A. U., Younas U., Rafique T., Khalid W., Ali Z., Nadeem M. (2024). Enhancement in the dielectric and magnetic properties of Ni 2+–Cu 2+ co-doped BaFe 11 Cu 1− x Ni x O 19 hexaferrites (0.0≤ x≤ 1.0). RSC Adv..

[cit24] Suresh P., Babu P., Srinath S. (2016). Role of (La, Gd) co-doping on the enhanced dielectric and magnetic properties of BiFeO3 ceramics. Ceram. Int..

[cit25] Chaurasiya S., Kumar S., Lone G. A., Kumar A., Sankhala K., Nazir N., Rashid A., Bhat S. A., Ikram M. (2024). Influence of Gd and Ni doping on the structural, morphological, and magnetic properties of M-type calcium hexaferrite. New J. Chem..

[cit26] Hench L. L., West J. K. (1990). The sol-gel process. Chem. Rev..

[cit27] Wahba M. A., Badawy A. A. (2020). Novel Zr–Cu–Fe nanocomposite metal oxides: structural, magnetic and composition activity effects on photodegradation of phenols. J. Sol–Gel Sci. Technol..

[cit28] Wahba M. A., Yakout S., Sheta S. M., Helal A., El-Sheikh S. M. (2024). Robust ferromagnetic V0. 05-XCoxZn0. 95O (x= 0.01, 0.02, 0.03, 0.04, 0.05) nano-compounds: New dilute magnetic-semiconductors with tailored optical activity. J. Alloys Compd..

[cit29] Zhao X., Wang X., Lin H., Wang Z. (2007). Electronic polarizability and optical basicity of lanthanide oxides. Phys. B.

[cit30] Gorbunov D., Henriques M., Andreev A., Eigner V., Gukasov A., Fabrèges X., Skourski Y., Petříček V., Wosnitza J. (2016). Magnetic anisotropy and reduced neodymium magnetic moments in N d 3 R u 4 A l 12. Phys. Rev. B.

[cit31] Wahba M. A., Khaled R. K. (2025). Sm/Gd and Sm/Gd/Nd co-doping for tailoring magnetic, dielectric, and optical properties in CoFe2O4 nanomaterials. J. Rare Earths.

[cit32] Townes W., Fang J., Perrotta A. (1967). The crystal structure and refinement of ferrimagnetic barium ferrite, BaFe12O19. Z. Kristallogr. Cryst. Mater..

[cit33] Sözeri H., Küçük I., Özkan H. (2011). Improvement in magnetic properties of La substituted BaFe12O19 particles prepared with an unusually low Fe/Ba molar ratio. J. Magn. Magn. Mater..

[cit34] Shannon R. D. (1976). Revised effective ionic radii and systematic studies of interatomic distances in halides and chalcogenides. Found. Crystallogr..

[cit35] Bindu P., Thomas S. (2014). Estimation of lattice strain in ZnO nanoparticles: X-ray peak profile analysis. J. Theor. Appl. Phys..

[cit36] Wahba M. A. (2025). Remarkable room-temperature ferromagnetic Zn0. 96Cu0. 04-xVx nanostructures: Novel diluted magnetic semiconductors with tailored optical properties. Mater. Chem. Phys..

[cit37] Fatimah S., Ragadhita R., Al Husaeni D. F., Nandiyanto A. B. D. (2022). How to calculate crystallite size from x-ray diffraction (XRD) using Scherrer method. ASEAN J. Sci. Eng..

[cit38] Wahba M. A., Mohamed W. A., Hanna A. A. (2016). Sol-gel synthesis, characterization of Fe/ZrO2 nanocomposites and their photodegradation activity on indigo carmine and methylene blue textile dyes. Int. J. ChemTech Res..

[cit39] Singh J., Singh C., Kaur D., Zaki H., Abdel-Latif I., Narang S. B., Jotania R., Mishra S. R., Joshi R., Dhruv P. (2017). Elucidation of phase evolution, microstructural, Mössbauer and magnetic properties of Co2+ Al3+ doped M-type BaSr hexaferrites synthesized by a ceramic method. J. Alloys Compd..

[cit40] Munnolli C. S., Kammar S., Barote V. K., Ibrahim A., Batoo K. M., Shirsath S. E., Kadam R., More S. (2024). Thorough investigation of functional properties of ferroelectric (Bi0. 5Na0. 5TiO3) 0.94-(BaTiO3) 0.06 and hexaferrite SrCe0. 05Sm0. 05 Fe11. 9O19 composites. J. Magn. Magn. Mater..

[cit41] Atkare S. S., Alone S. T., Fulari A. V., Dhage V. N., Kadam R., Shirsath S. E., Mane M. L. (2025). Design of multifunctional rare earth Dy–Ce substituted BaFe12O19 nanoparticles for X-band microwave absorption and EMI shielding. Inorg. Chem. Commun..

[cit42] Chawla S., Mudsainiyan R., Meena S., Yusuf S. (2014). Sol–gel synthesis, structural and magnetic properties of nanoscale M-type barium hexaferrites BaCoxZrxFe (12− 2x) O19. J. Magn. Magn. Mater..

[cit43] Wang H., Su K., Huang S., Tan W. (2017). J. Mater. Sci.: Mater. Electron..

[cit44] Wahba M. A., Yakout S. M., Elprince A., Abdel-Monem Y. K. (2025). Perovskite Gd/Nd/Fe-BaSnO3 semiconductors: Enhanced ferromagnetic order for advanced electronic-chips. Mater. Chem. Phys..

[cit45] Badry M. D., Wahba M. A., Khaled R. K., Moawad M. (2021). Hydrothermally assisted synthesis of magnetic iron oxide-chitosan nanocomposites: Electrical and biological evaluation. Challenge.

[cit46] Wahba M. A. (2025). Unveiling significant changes in optical, magnetic, and visible-light photocatalytic performance of CuFe2O4 nanocompositions through chelating agent modulation. Ceram. Int..

[cit47] Taran M. N., Dyar M. D., Matsyuk S. S. (2007). Optical absorption study of natural garnets of almandine-skiagite composition showing intervalence Fe2++ Fe3+→ Fe3++ Fe2+ charge-transfer transition. Am. Mineral..

[cit48] Sherman D. M., Waite T. D. (1985). Electronic spectra of Fe3+ oxides and oxide hydroxides in the near IR to near UV. Am. Mineral..

[cit49] Kubelka P., Munk F. (1931). A contribution to the optics of pigments. Z. Tech. Phys..

[cit50] Wahba M. A. (2024). Visible-light responsive BiVO4 nanocompositions: Enhanced photocatalytic, electrical and optical performance through Ni and Ni/Co doping. Opt. Mater..

[cit51] Wahba M. A., Yakout S. M., Youssef A., Sharmoukh W., sayed A. E., Khalil M. S. (2022). Chelating agents assisted rapid synthesis of high purity BiFeO3: remarkable optical, electrical, and magnetic characteristics. J. Supercond. Novel Magn..

[cit52] Wahba M. A., Khaled R. K. (2025). V/Zn Incorporated-MCM-41: synthesis, characterization and visible light photocatalytic activity for Tetracycline degradation. J. Inorg. Organomet. Polym. Mater..

[cit53] Kamali A., Hasani S., Hashemi S. M., Soltani-Nezhad S., Rezvan M. T., Seifoddini A., Nouri-Khezrabad M. (2025). Structural, magnetic, and optical properties of barium hexaferrite synthesized via albumin-aided co-precipitation: Taguchi-optimized experimental and DFT computational studies. J. Alloys Compd..

[cit54] Chaudhary H., Ganesan S., Pathak P. K., Ramesh M. D., Ahmed J., Aich S., Verma A., Kit C. C., Lakshmaiya N., Jasrotia R. (2024). Robustic and hybrid cobalt doped BaFe12O19 hexaferrites for the photocatalytic degradation of Congo Red for wastewater treatment. Sci. Rep..

[cit55] Mousavi M., Habibi-Yangjeh A., Abitorabi M. (2016). Fabrication of novel magnetically separable nanocomposites using graphitic carbon nitride, silver phosphate and silver chloride and their applications in photocatalytic removal of different pollutants using visible-light irradiation. J. Colloid Interface Sci..

[cit56] Morrison S. R. and Morrison S., Electrochemistry at Semiconductor and Oxidized Metal Electrodes, Springer, 1980, vol. 126

[cit57] Tripathy S. (2015). Refractive indices of semiconductors from energy gaps. Opt. Mater..

[cit58] Mehrabani M., Ghazi M. E., Izadifard M., Morales I., Johannes H.-H., Kowalsky W. (2025). Effect of co-doped Zn2+- Ni2+ on the structural, optical and magnetic properties of BaFe12O19 hexaferrite. J. Alloys Compd..

[cit59] Alikhanov N., Rabadanova A., Magomedova A., Abdulkerimov M., Gyulakhmedov R., Shuaibov A., Selimov D., Sobola D., Ramazanov S., Abdulvakhidov K. (2025). Structural, Dielectric, Magnetic and Sonophotocatalytic Properties of M-Type BaFe12O19 Hexaferrite Synthesized by Solution Combustion Method. Surf. Interfaces.

[cit60] Tannous C., Gieraltowski J. (2008). The Stoner–Wohlfarth model of ferromagnetism. Eur. J. Phys..

[cit61] Islam M. R., Khan M., Hossain M. S., Rahman M., Haque M. M., Aliuzzaman M., Alam M., Sarker M. (2024). Structural, thermodynamic, and magnetic properties of SrFe 12 O 19 hexaferrite modified by co-substitution of Cu and Gd. RSC Adv..

[cit62] Das A., Roychowdhury A., Pati S., Bandyopadhyay S., Das D. (2015). Structural, magnetic and hyperfine properties of single-phase SrFe12O19 nanoparticles prepared by a sol–gel route. Phys. Scr..

