# Supplementary material for: Synergistic tailoring of structural, optical, dielectric, and magnetic responses in Ce/Ni- and Nd/Ni-co-doped M-type BaFe12O19 nano-compositions
Source: RSC Adv. 2026 Aug 10;16(40):46439–57. doi: 10.1039/d6ra03457h (PMC13456046; doi:10.1039/d6ra03457h)
Supplement: Supplementary file 1 [file RA-016-D6RA03457H-s001.pdf]

Supplementary Information (SI) for RSC Advances.
This journal is © The Royal Society of Chemistry 2026

Supplementary Materials for:

Synergistic tailoring of structural, optical, dielectric, and magnetic responses in Ce/Ni- and Nd/Ni-co-doped M-type $BaFe_{12}O_{19}$ Nano-compositions

This supplementary file provides additional FESEM images, particle size distributions, EDS spectra, and elemental mapping corresponding to the BF, CNBF, and NNBF samples discussed in the main manuscript.

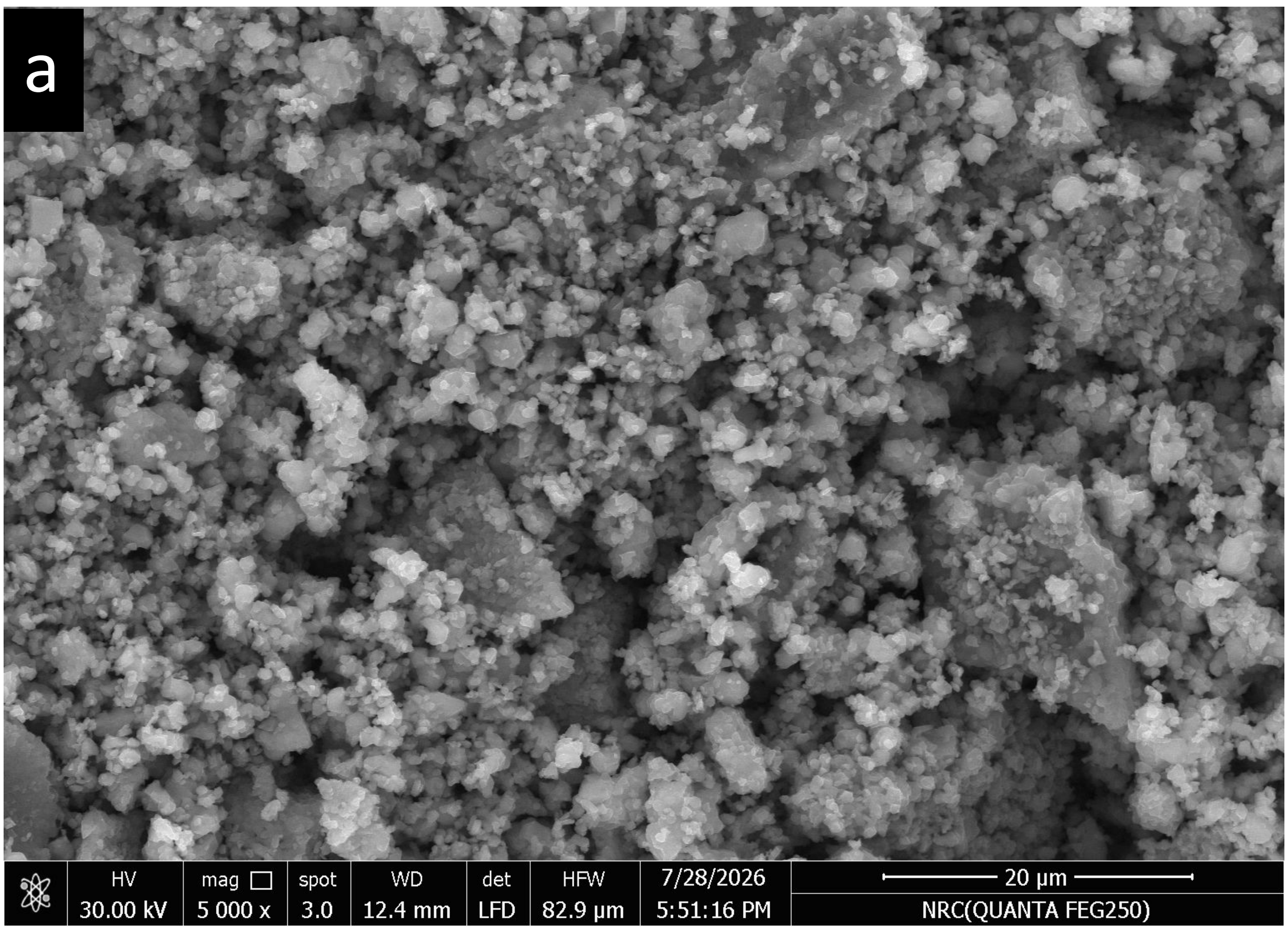

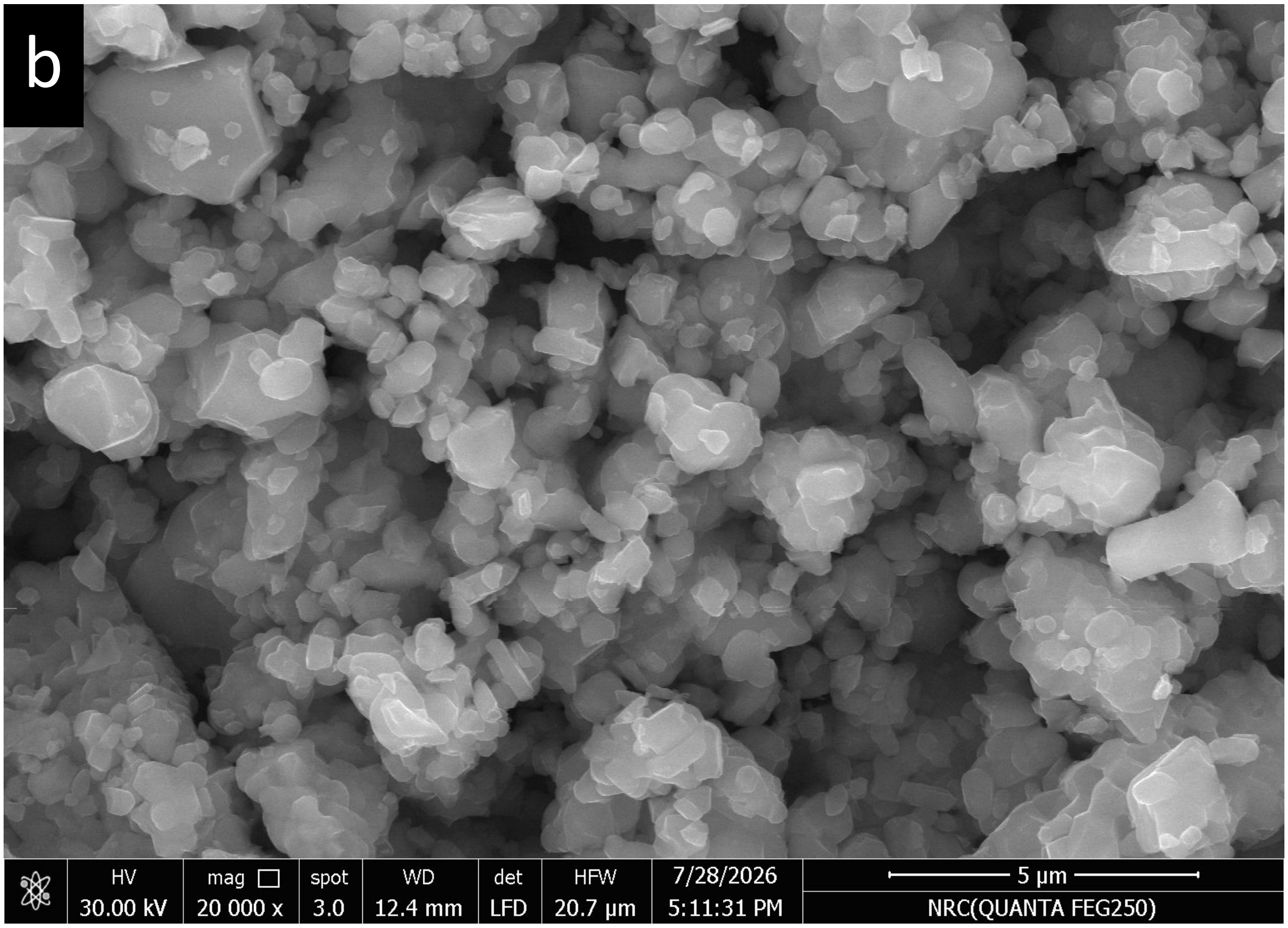

b
HV
30.00 kV
mag
20 000 x
spot
3.0
WD
12.4 mm
det
LFD
HFW
20.7 µm
7/28/2026
5:11:31 PM
5 µm
NRC(QUANTA FEG250)

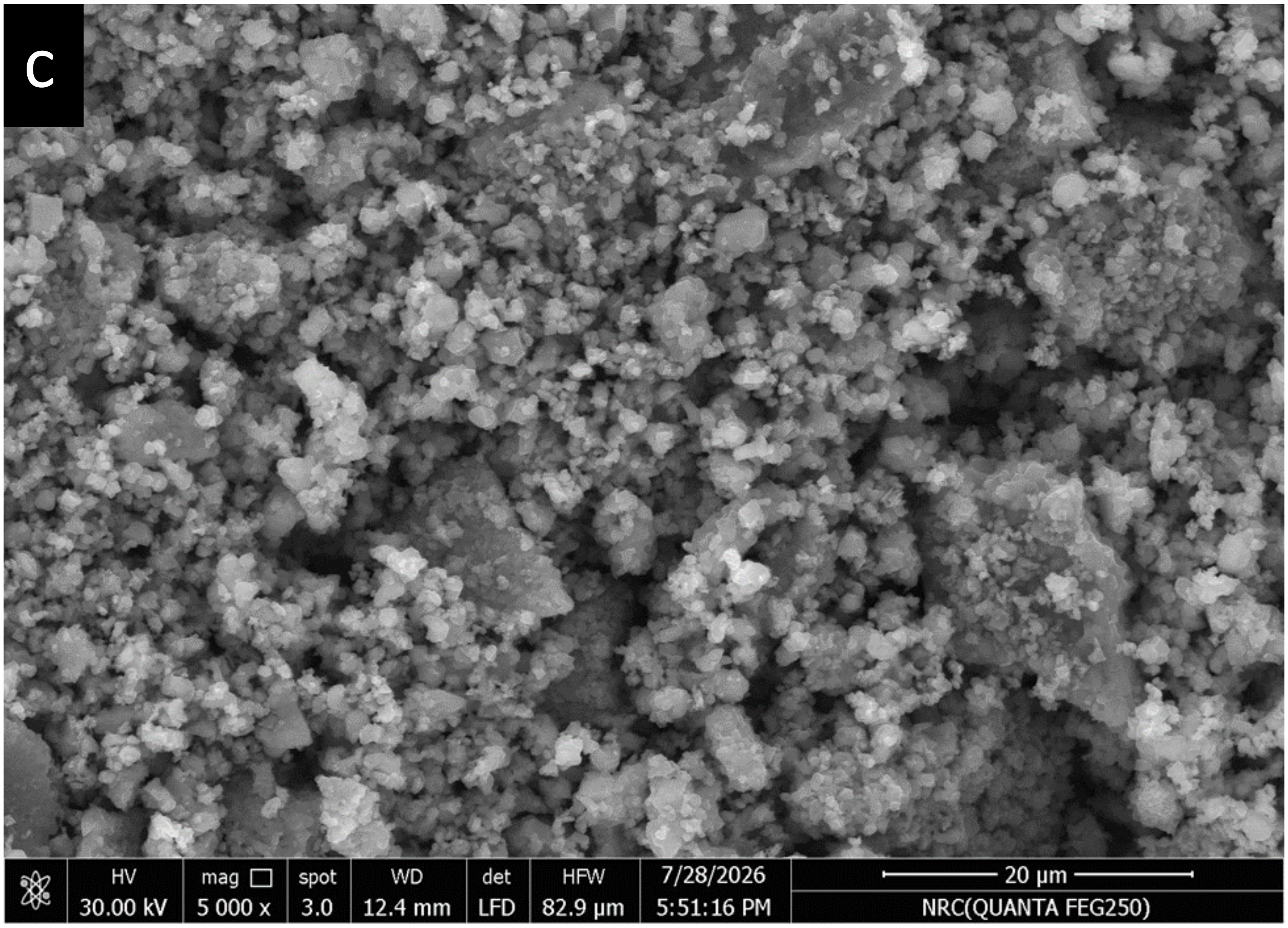

c
HV
30.00 kV
mag
5 000 x
spot
3.0
WD
12.4 mm
det
LFD
HFW
82.9 µm
7/28/2026
5:51:16 PM
20 µm
NRC(QUANTA FEG250)

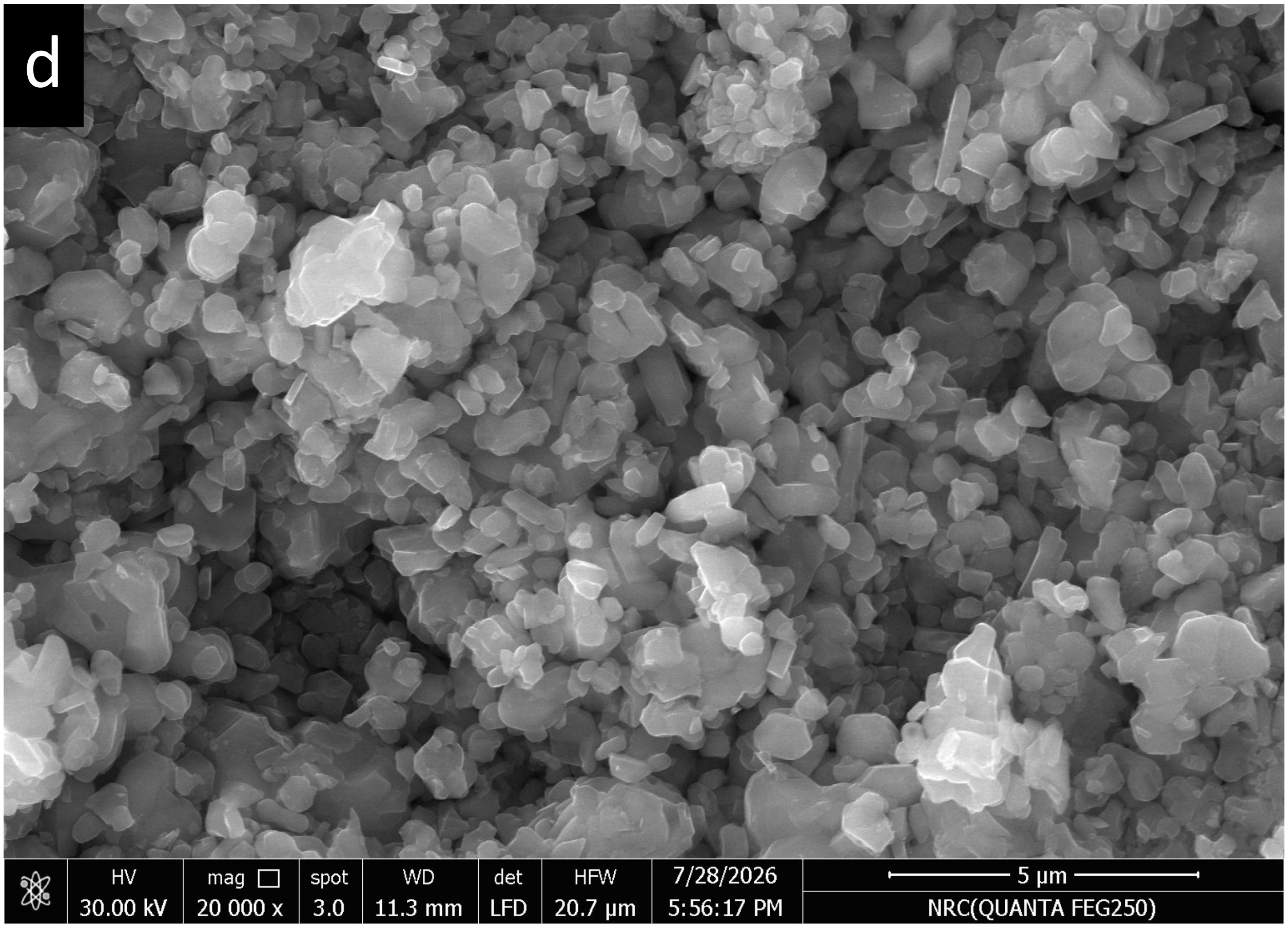
d
HV
30.00 kV
mag
20 000 x
spot
3.0
WD
11.3 mm
det
LFD
HFW
20.7 µm
7/28/2026
5:56:17 PM
5 µm
NRC(QUANTA FEG250)

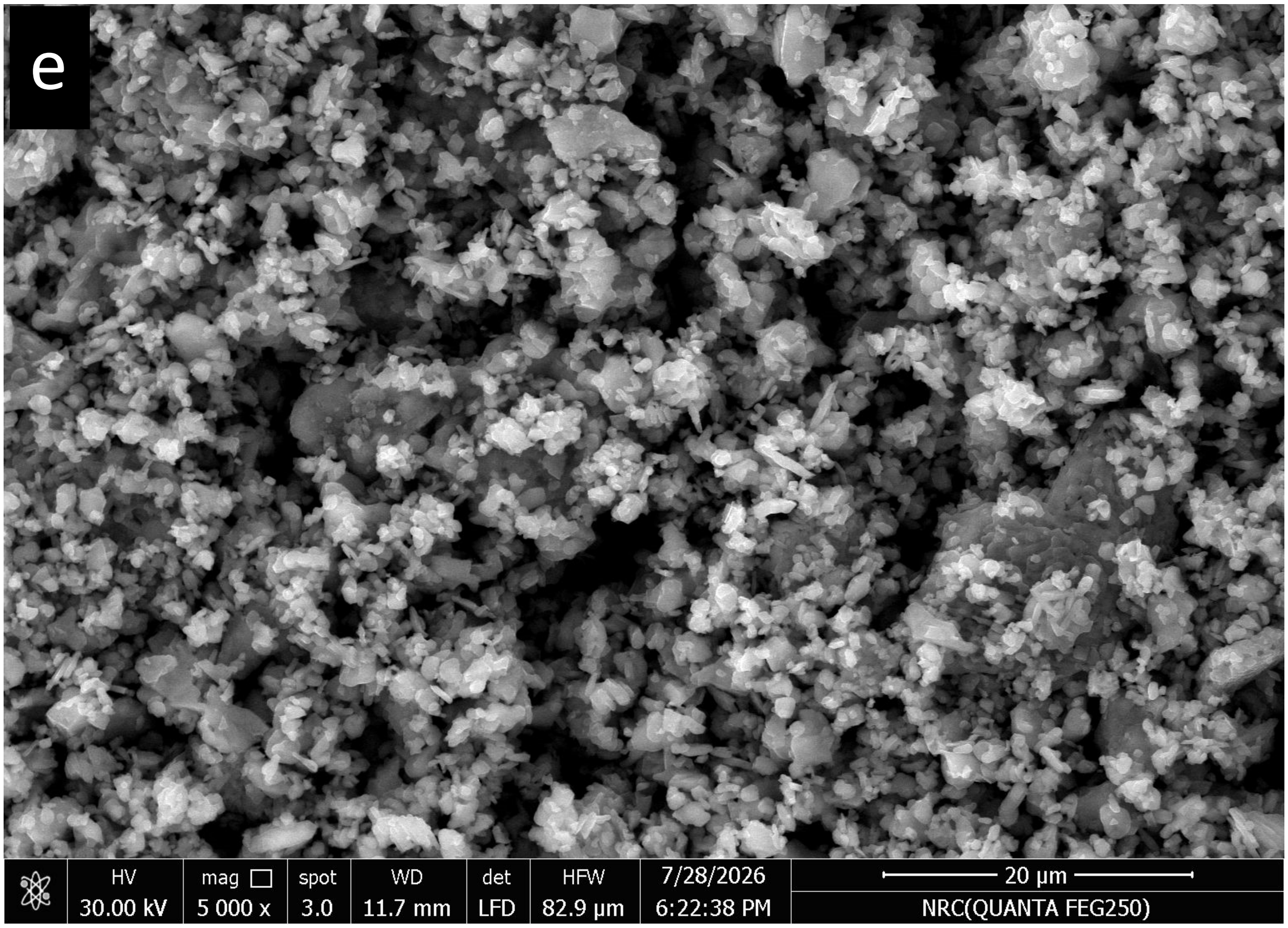
e
HV
30.00 kV
mag
5 000 x
spot
3.0
WD
11.7 mm
det
LFD
HFW
82.9 µm
7/28/2026
6:22:38 PM
20 µm
NRC(QUANTA FEG250)

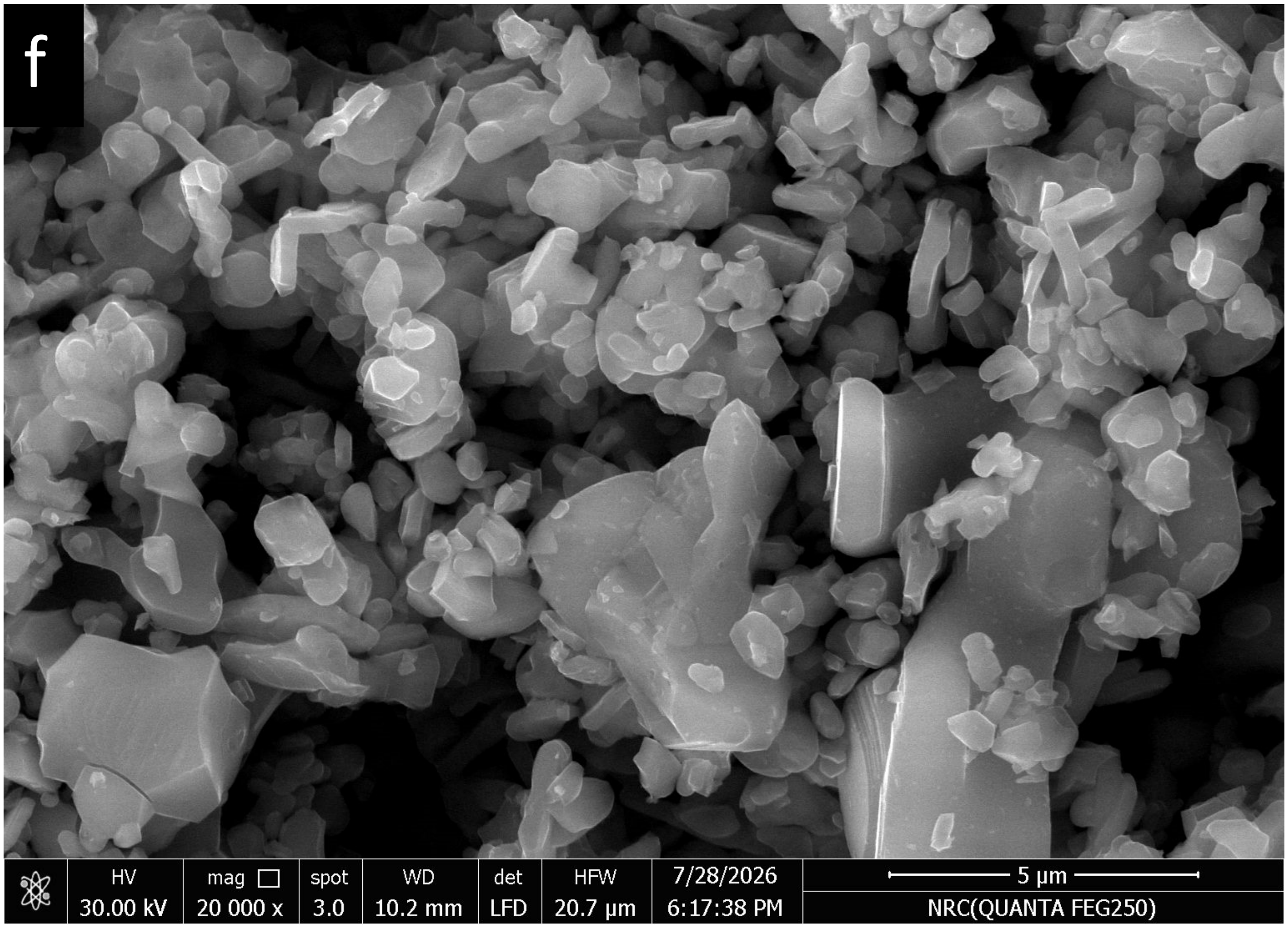

Figure S1. FESEM micrographs of (a,b) BF, (c,d) CNBF, and (e,f) NNBF samples acquired at 5,000× and 20,000× magnifications, respectively.

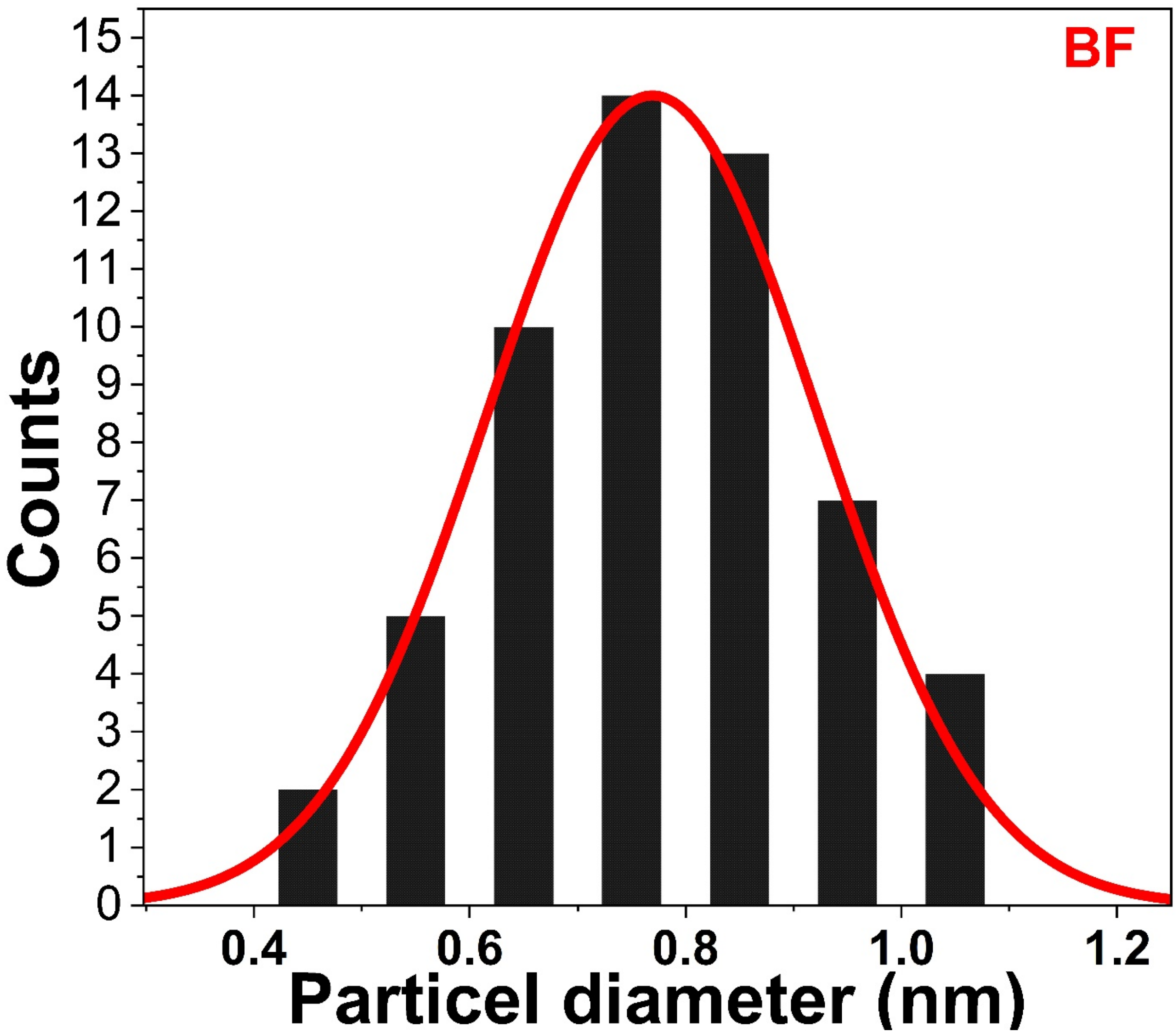

BF
Counts
Particel diameter (nm)
0
1
2
3
4
5
6
7
8
9
10
11
12
13
14
15
0.4
0.6
0.8
1.0
1.2

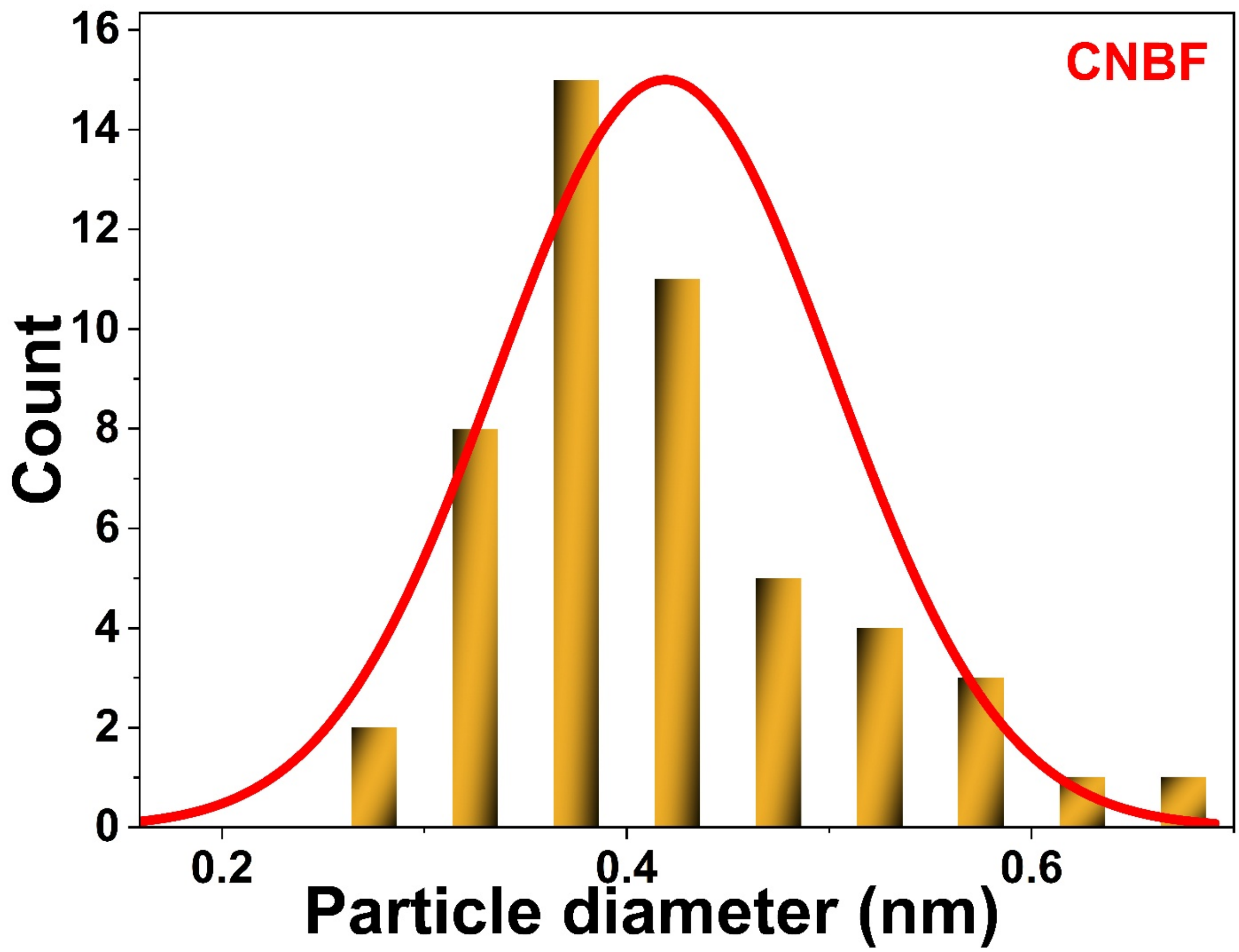

CNBF
Count
Particle diameter (nm)
0
2
4
6
8
10
12
14
16
0.2
0.4
0.6

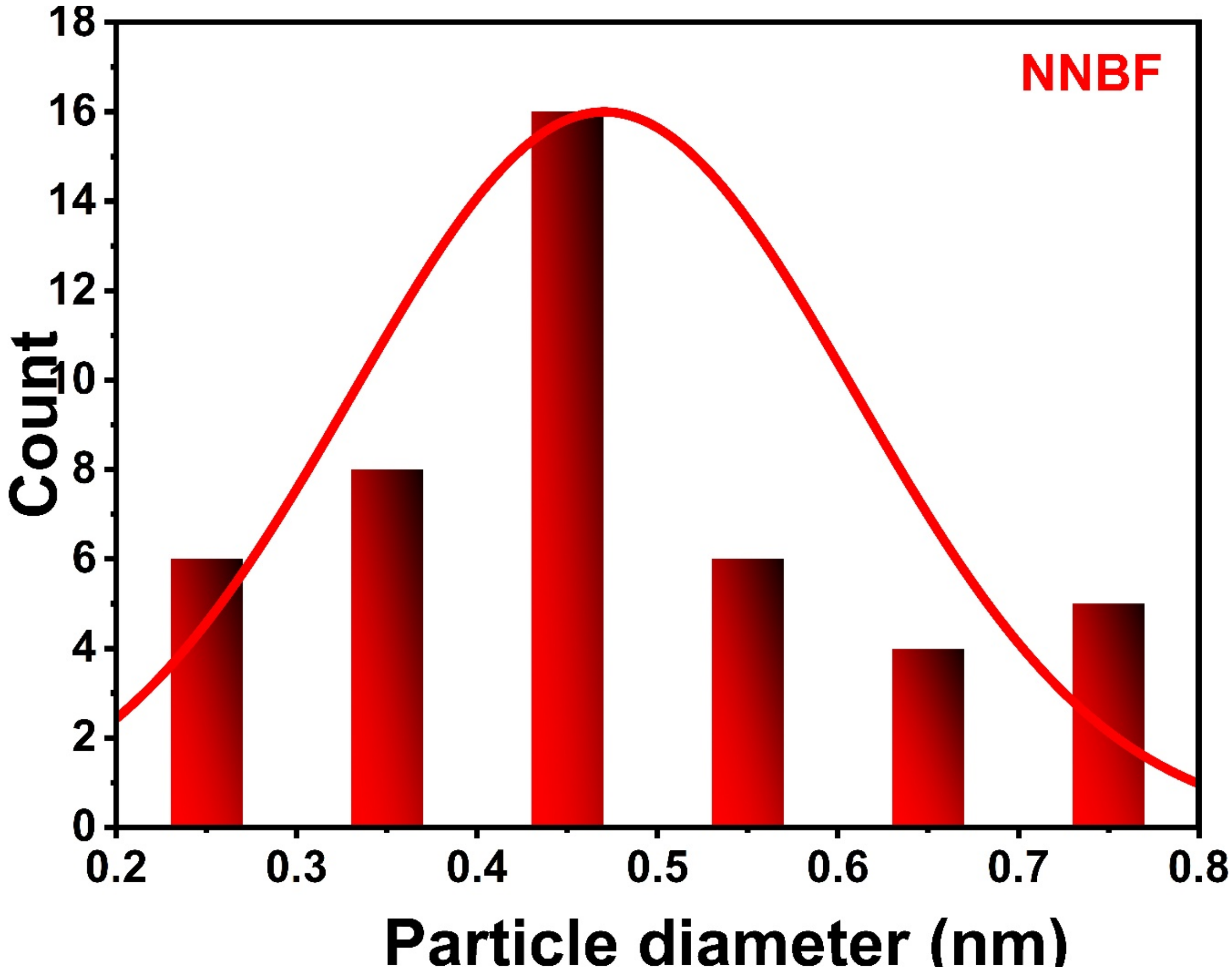

Figure S.2: Particle size distribution of BF, CNBF and NNBF samples.

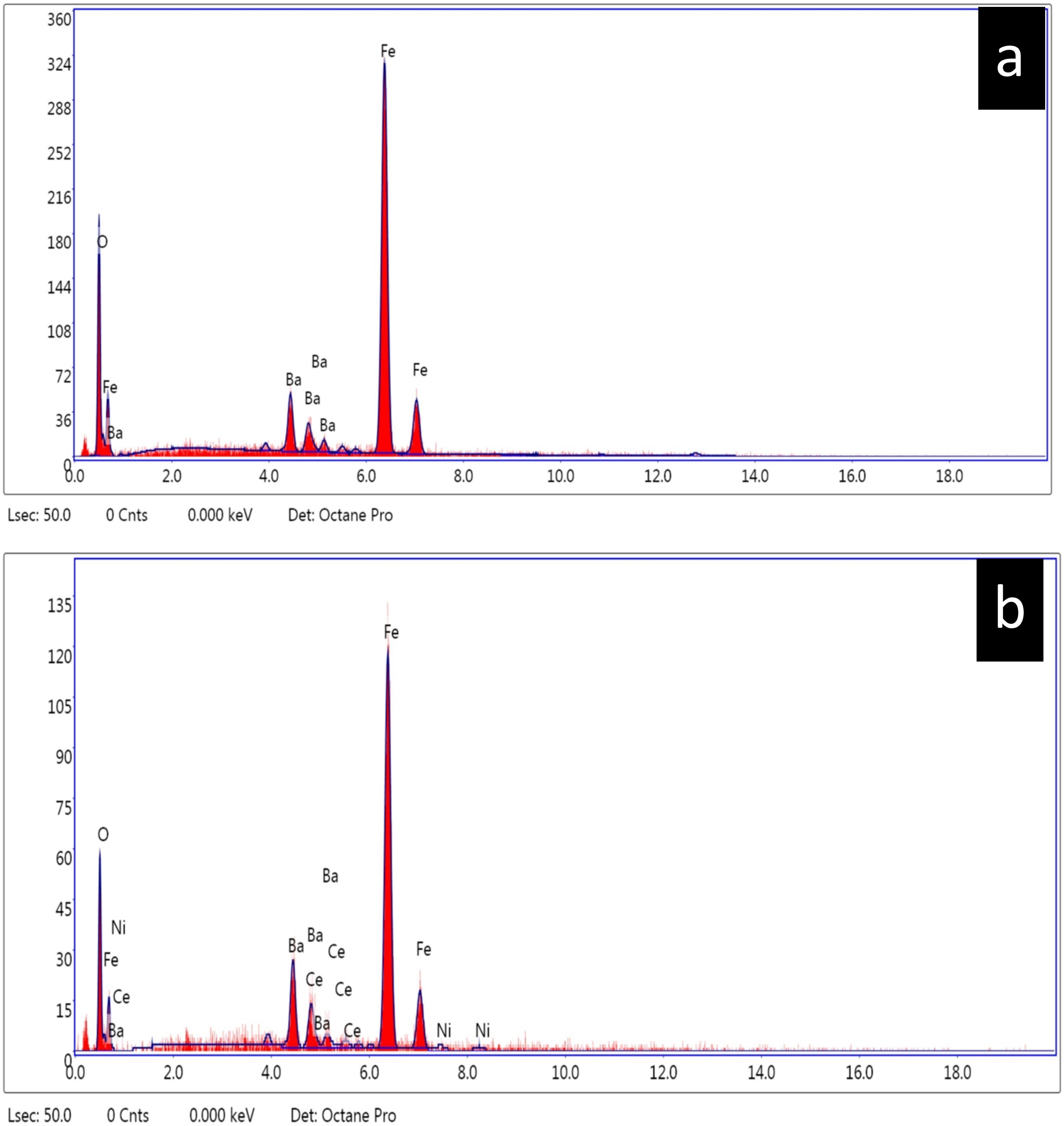

Figure S3. EDX spectra of BF and CNBF samples confirming the elemental composition of the synthesized materials.

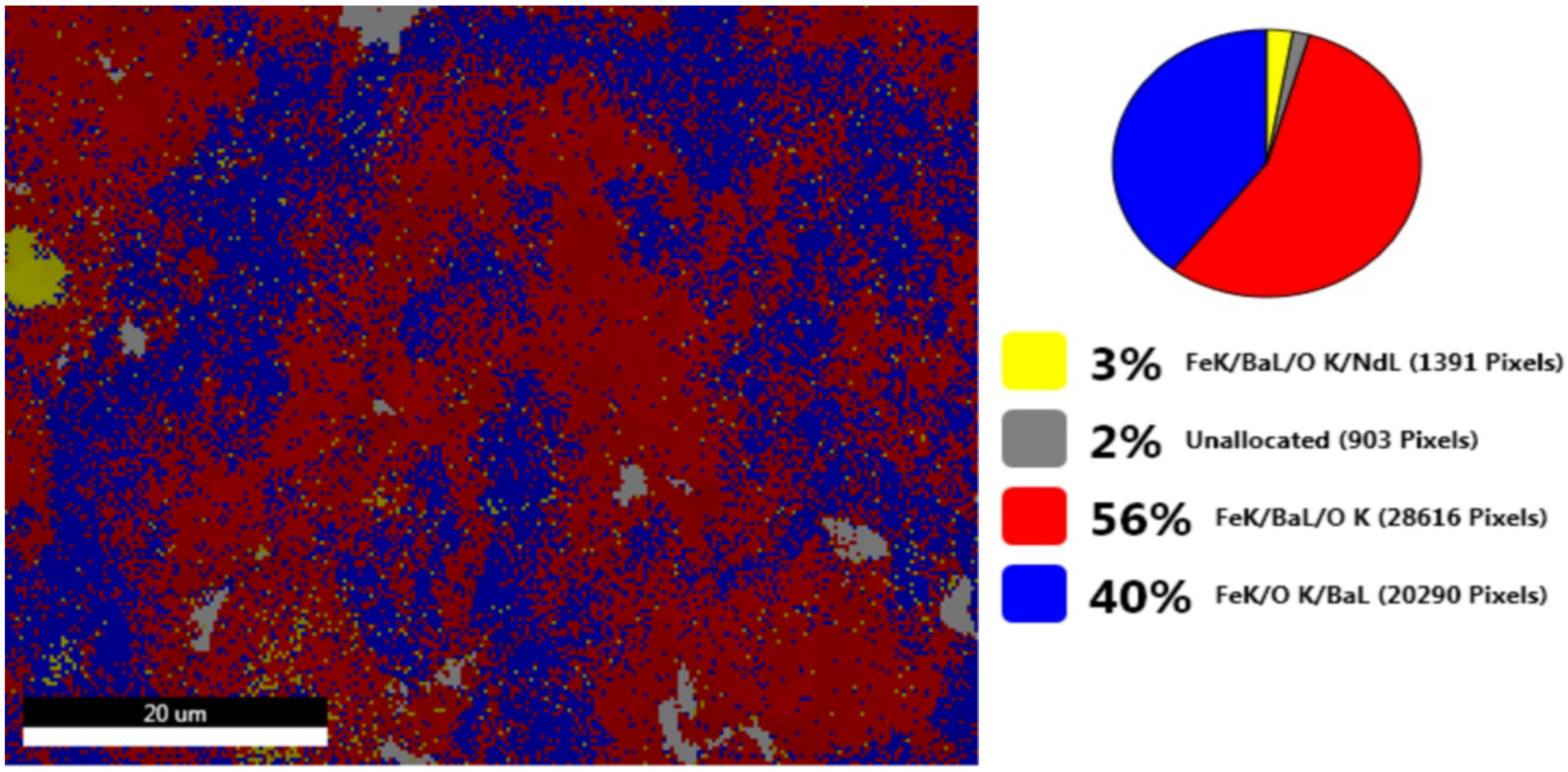
20 um
3% FeK/BaL/O K/NdL (1391 Pixels)
2% Unallocated (903 Pixels)
56% FeK/BaL/O K (28616 Pixels)
40% FeK/O K/BaL (20290 Pixels)

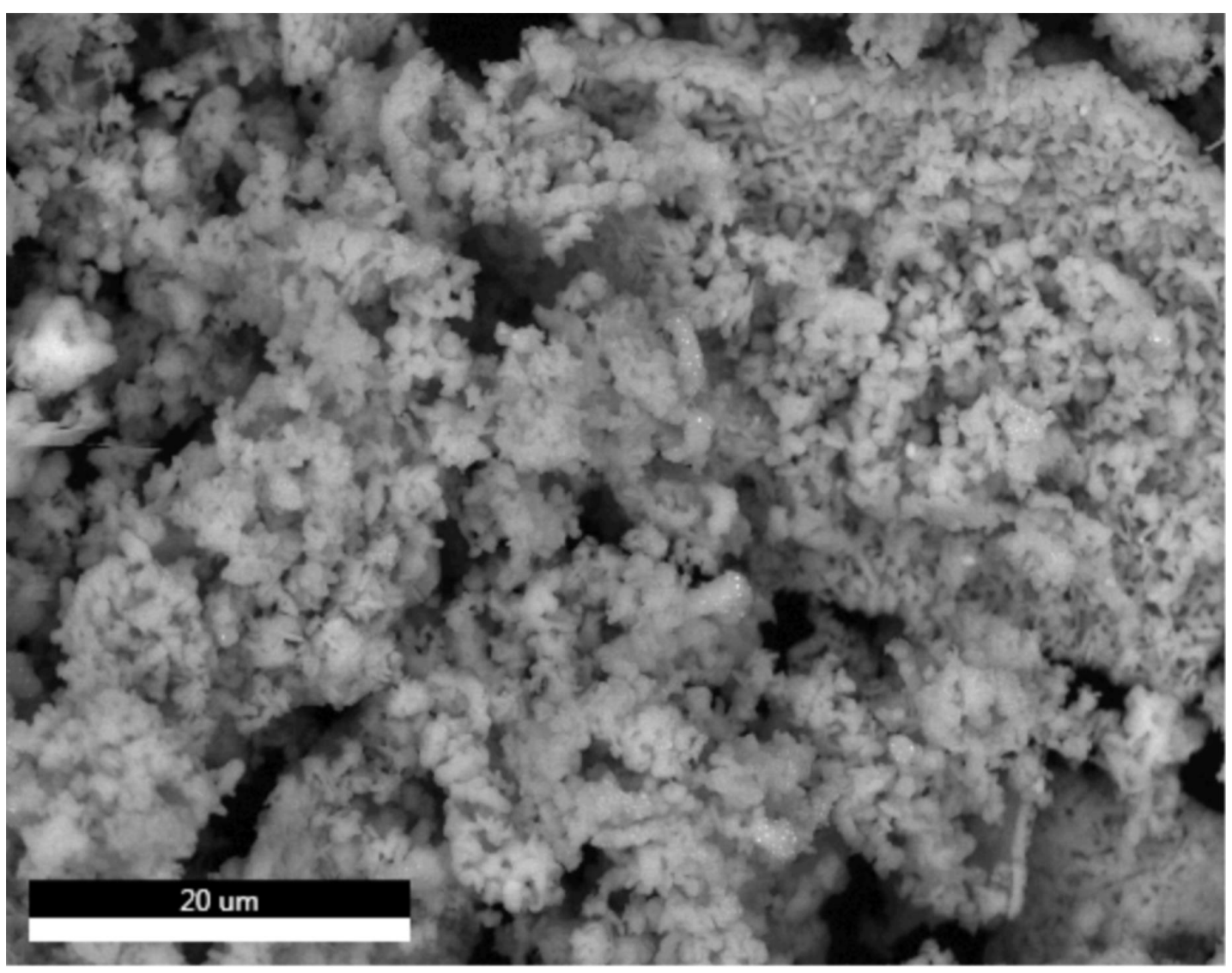
20 um

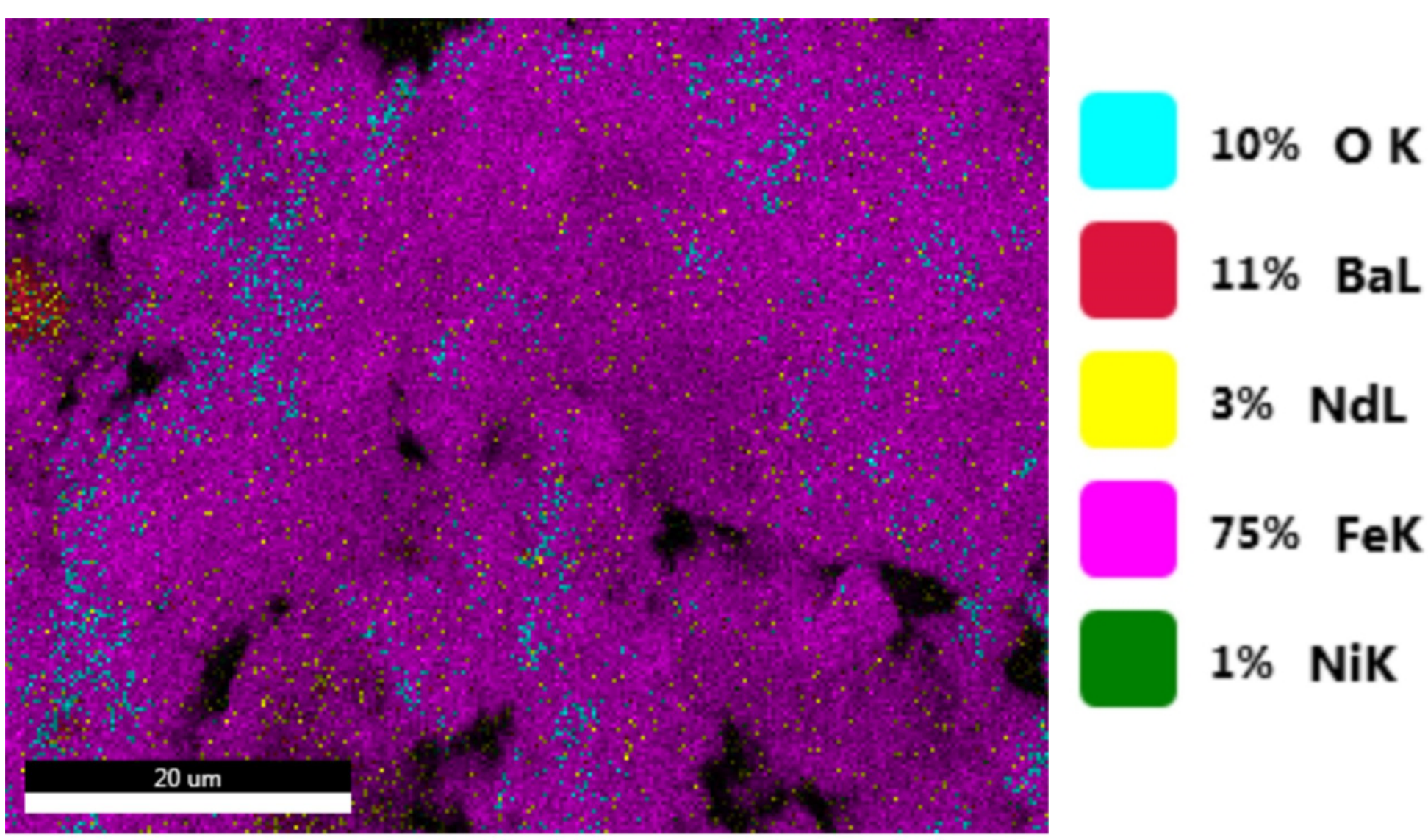
20 um
10% O K
11% BaL
3% NdL
75% FeK
1% NiK

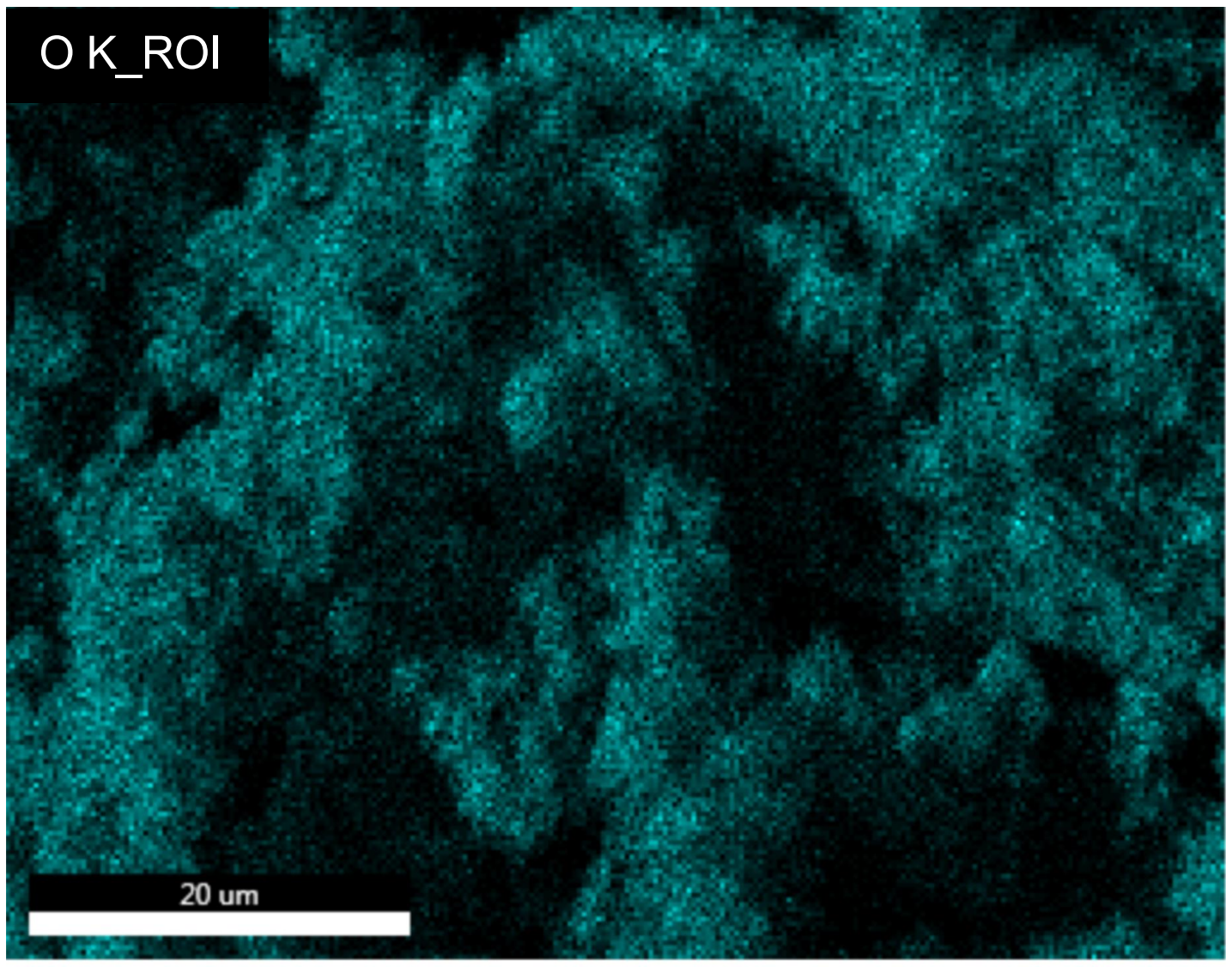
O K_ROI
20 um

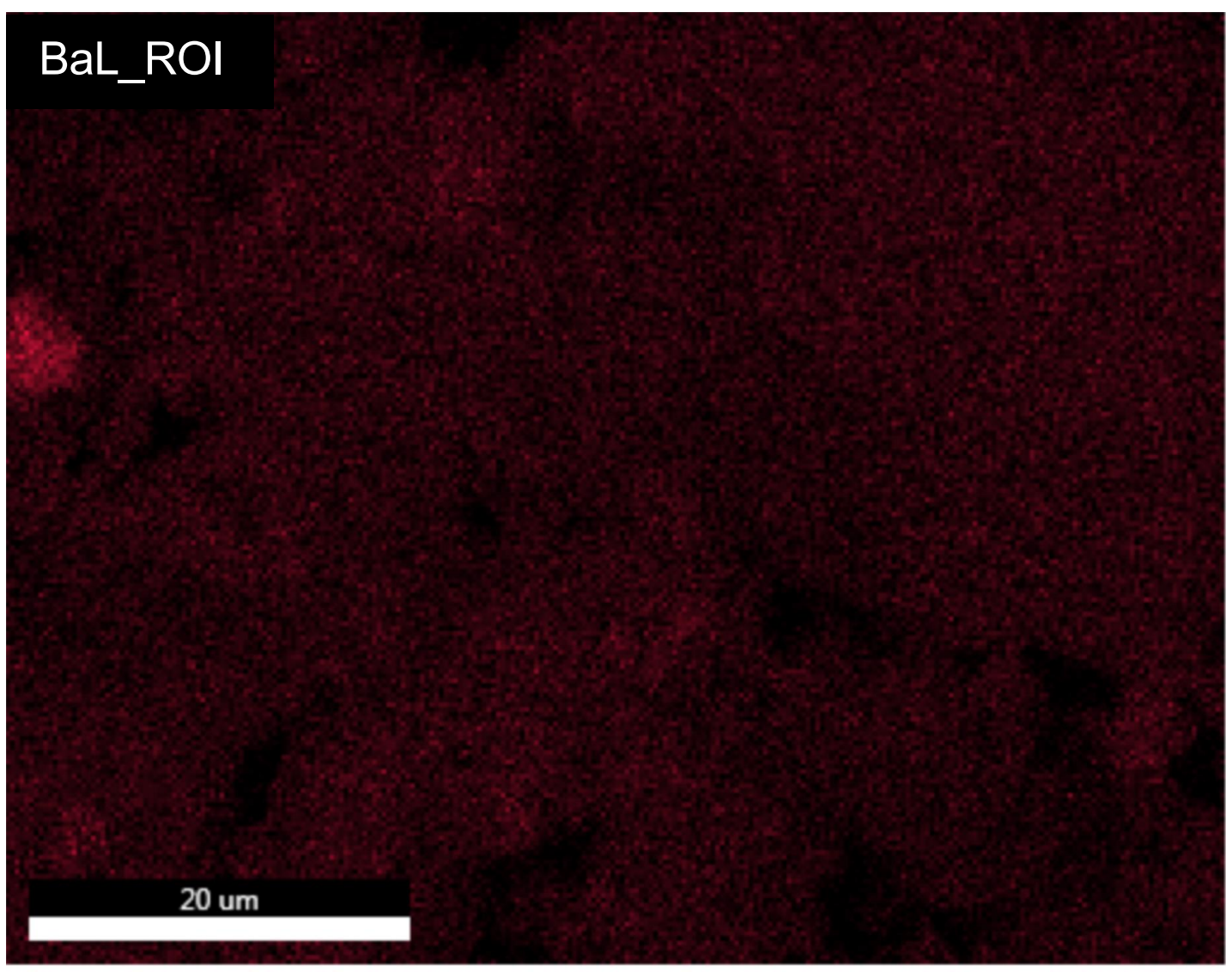
BaL_ROI
20 um

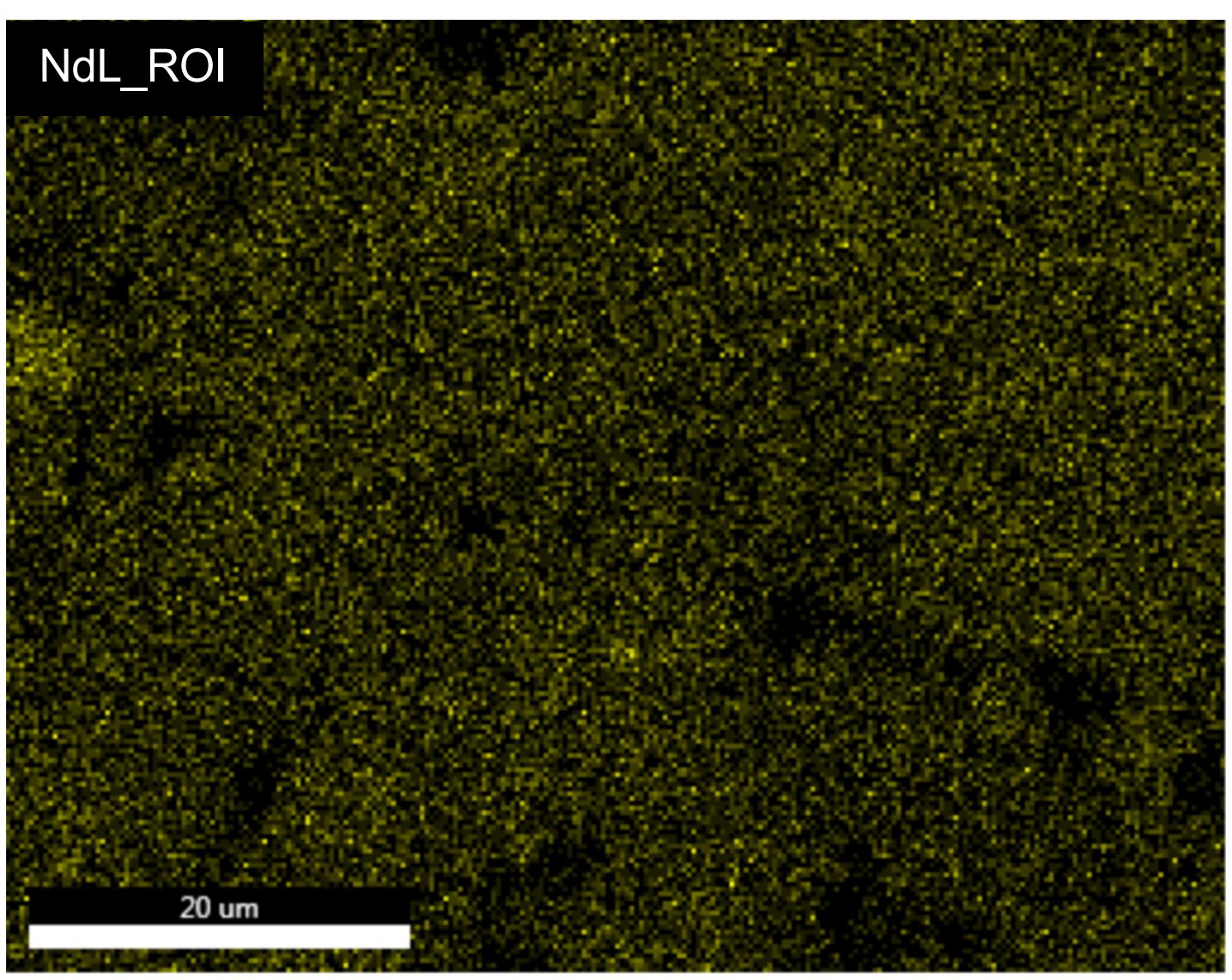
NdL_ROI
20 um

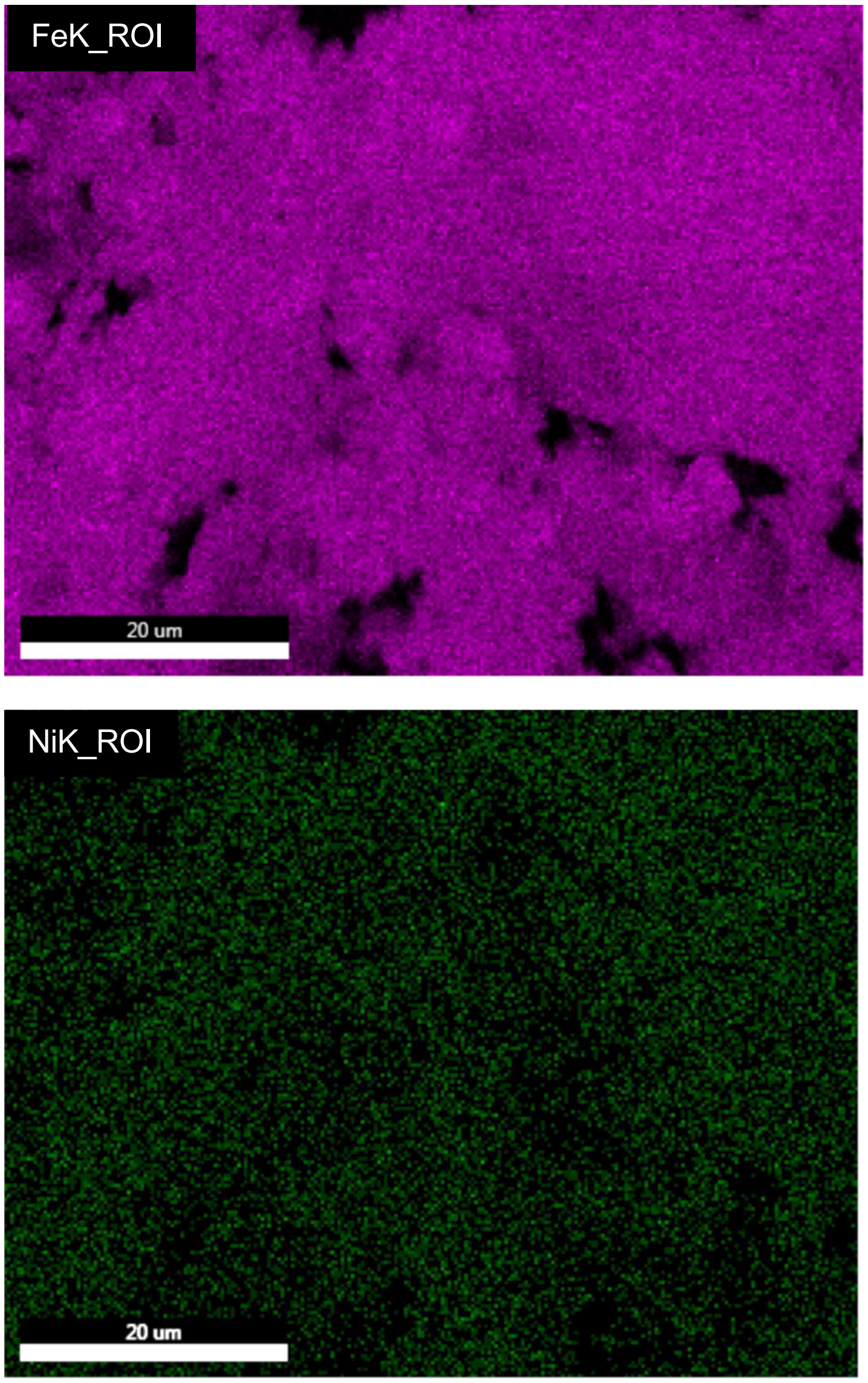

Figure S4. Elemental mapping images of the NNBF sample showing the homogeneous spatial distribution of Ba, Fe, O, Nd, and Ni.

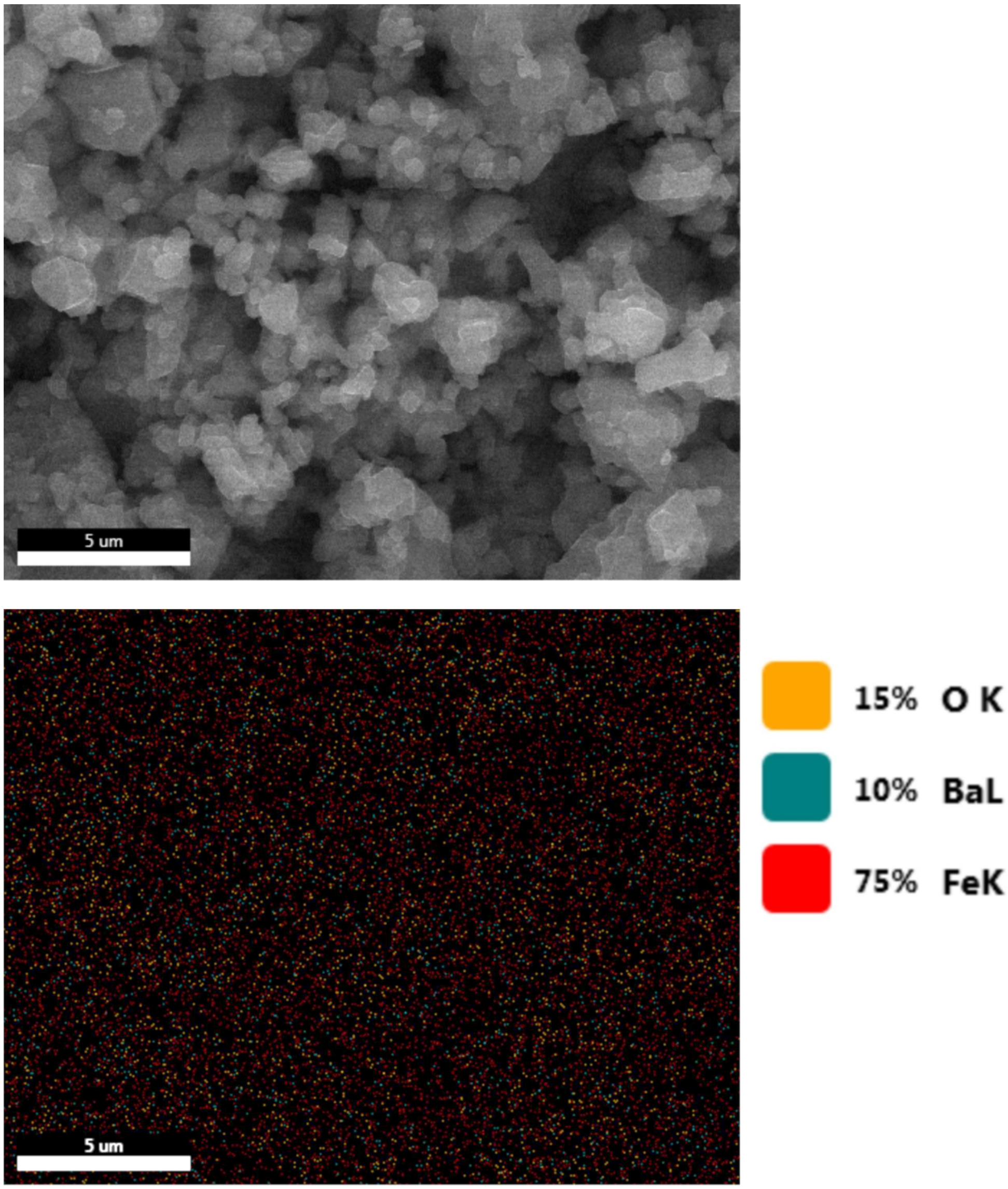
5 um
5 um
15% O K
10% BaL
75% FeK

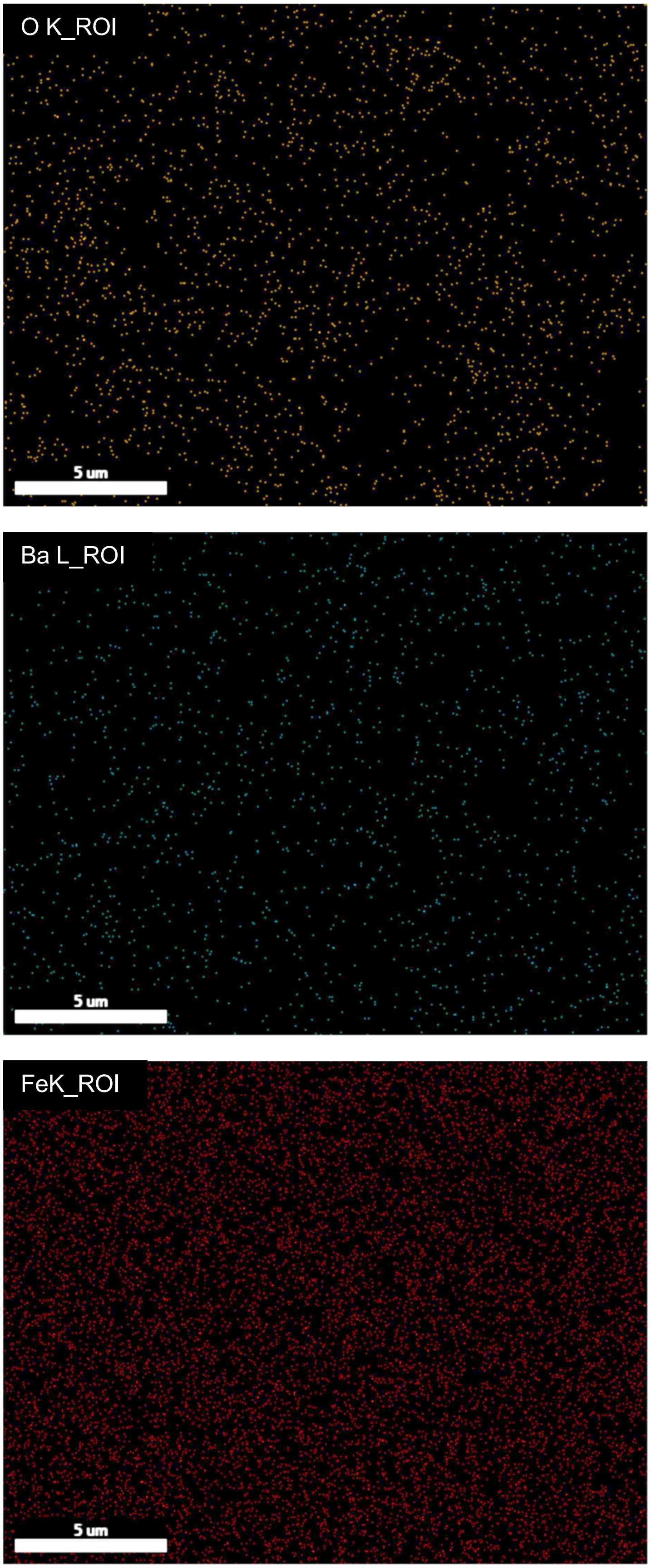
O K_ROI
5 um
Ba L_ROI
5 um
FeK_ROI
5 um

Figure S.5: Elemental mapping images of BF sample showing the spatial distribution of Ba, Fe, and O throughout the sample.

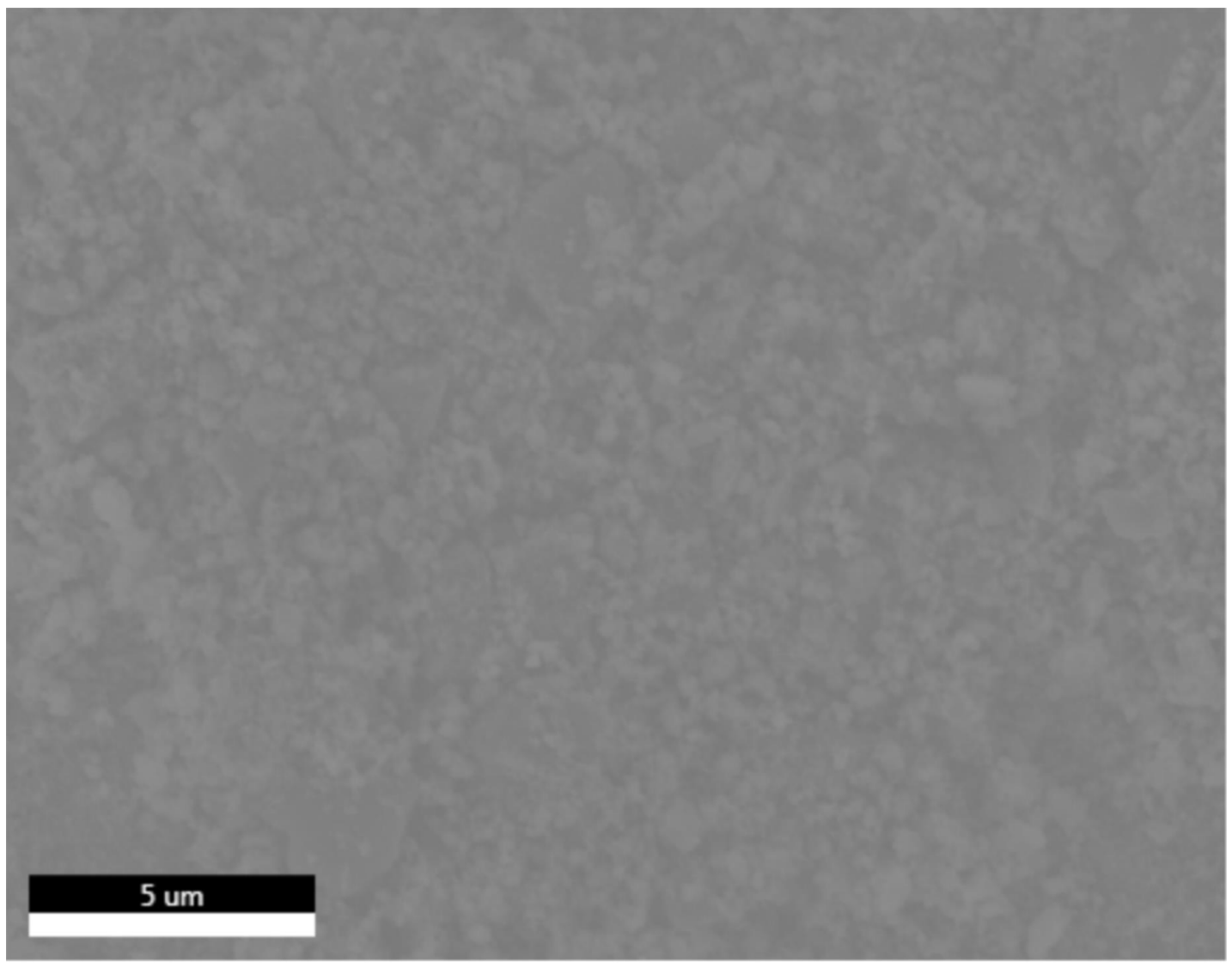

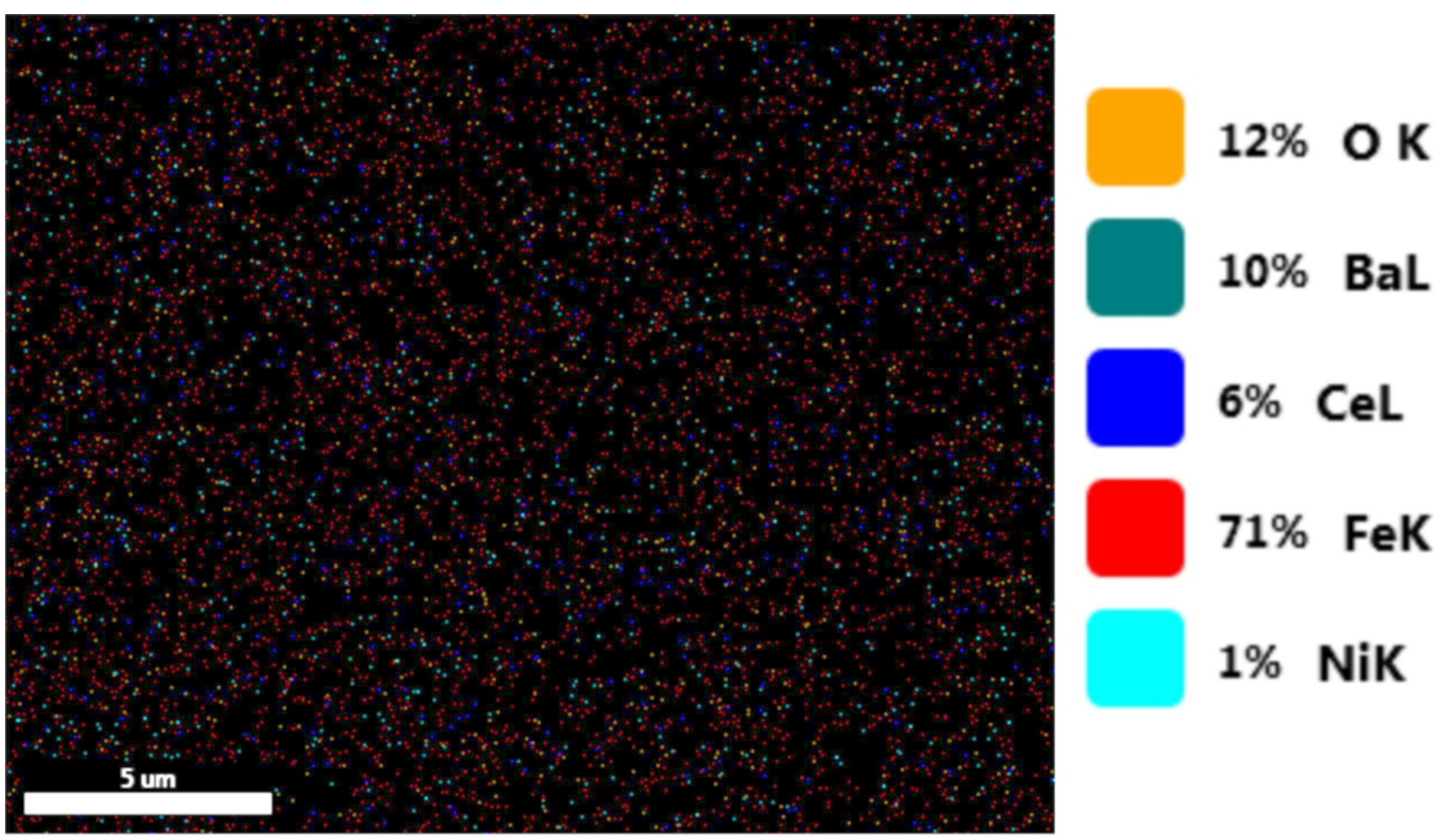

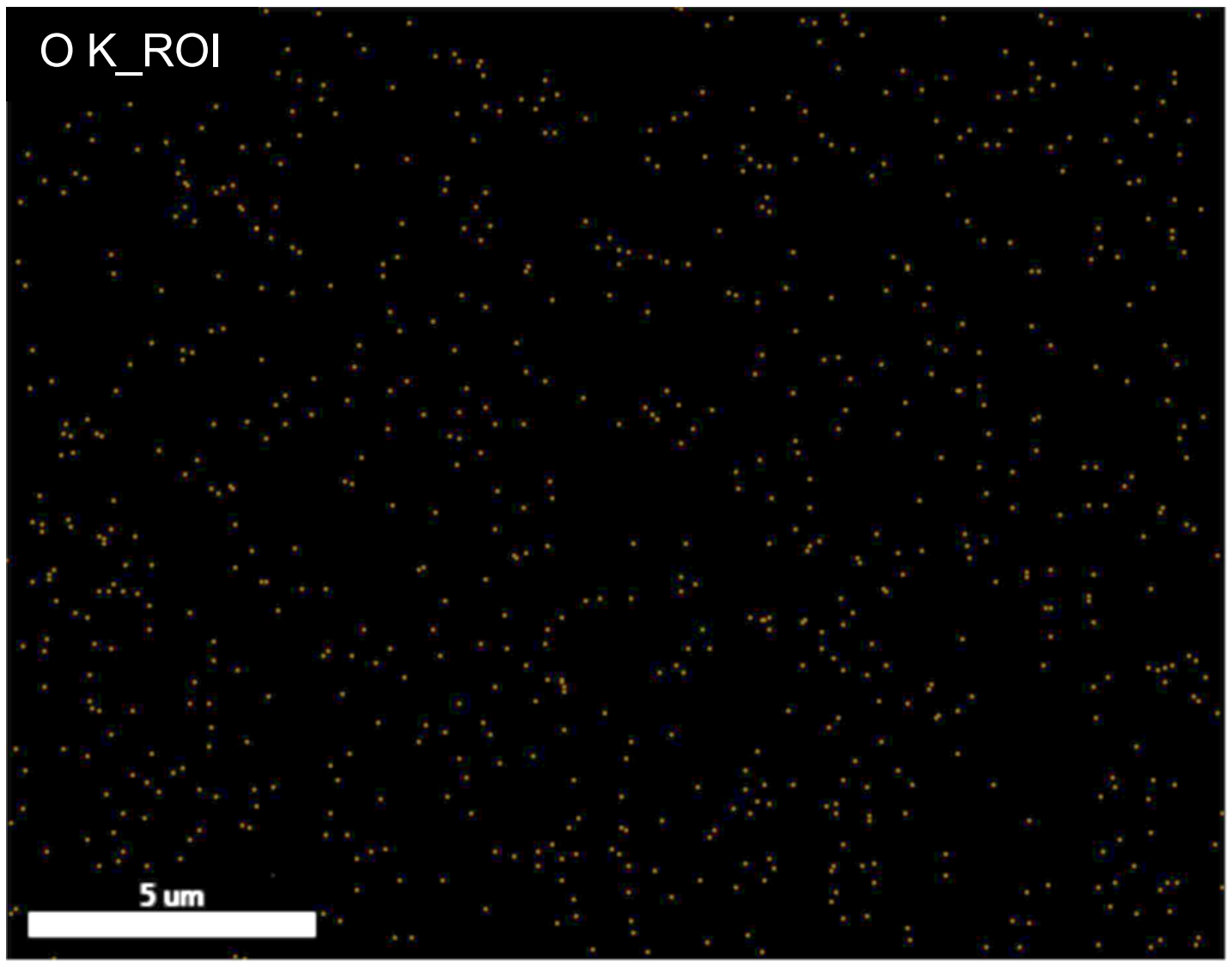
O K_ROI
5 um

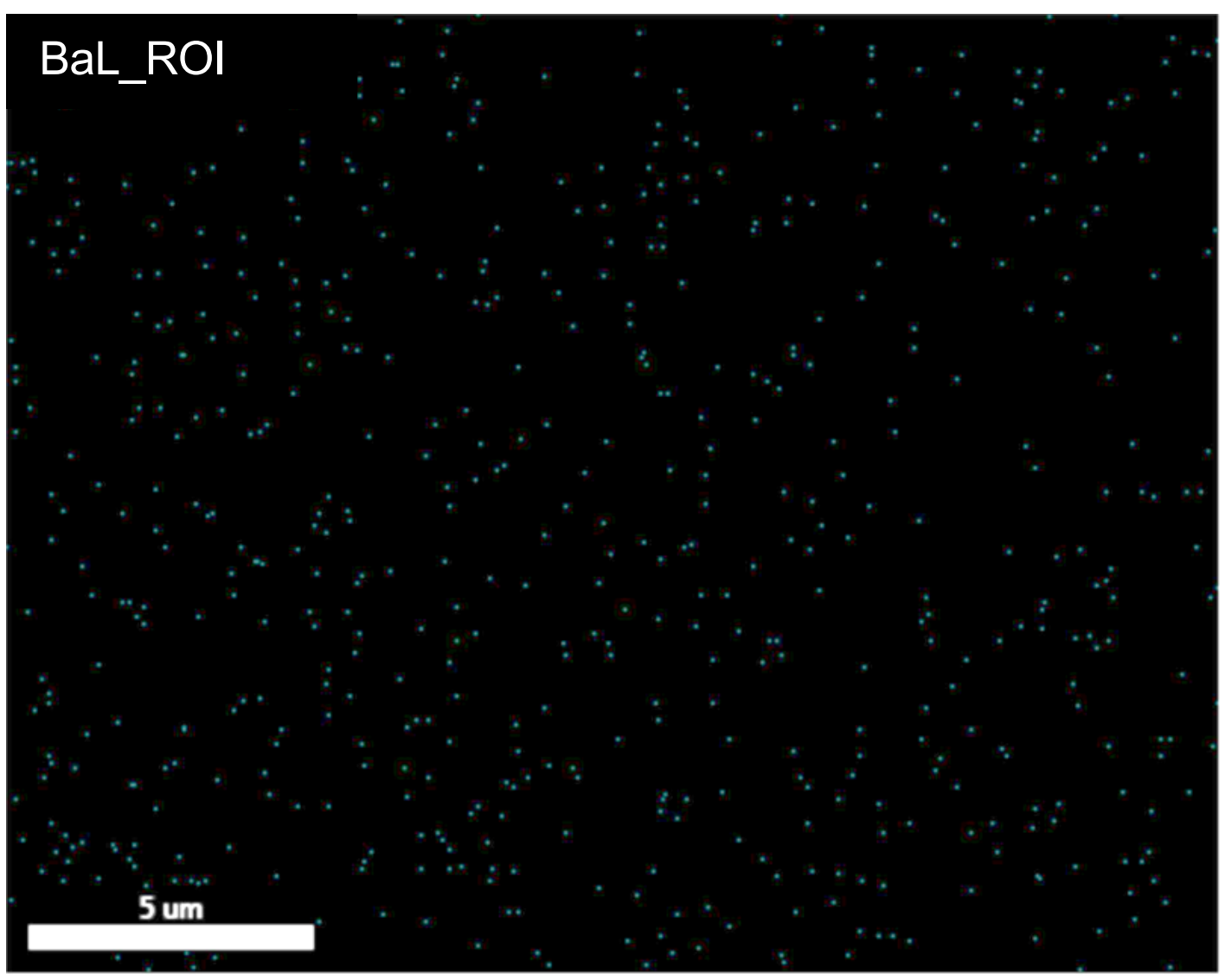
BaL_ROI
5 um

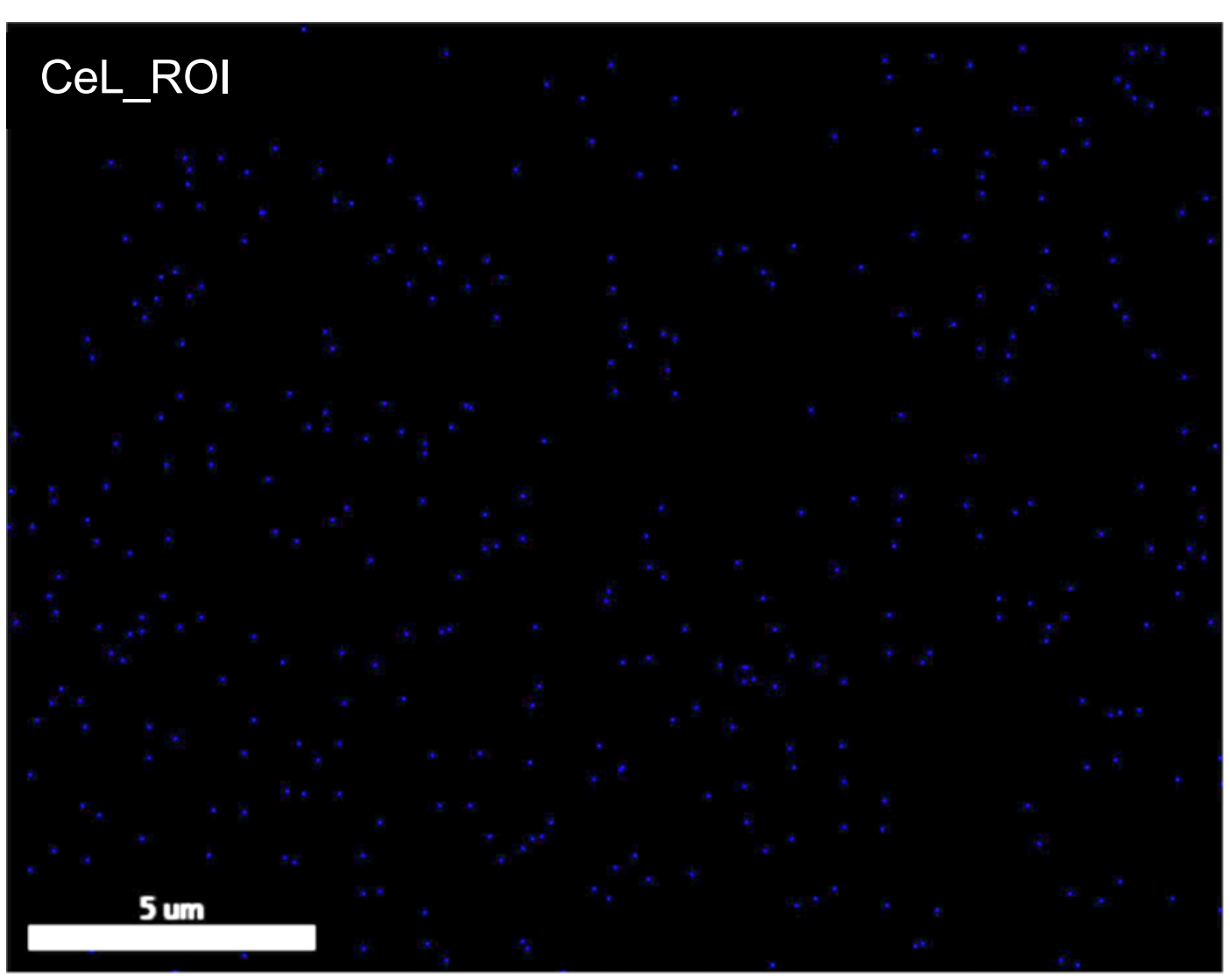
CeL_ROI
5 um

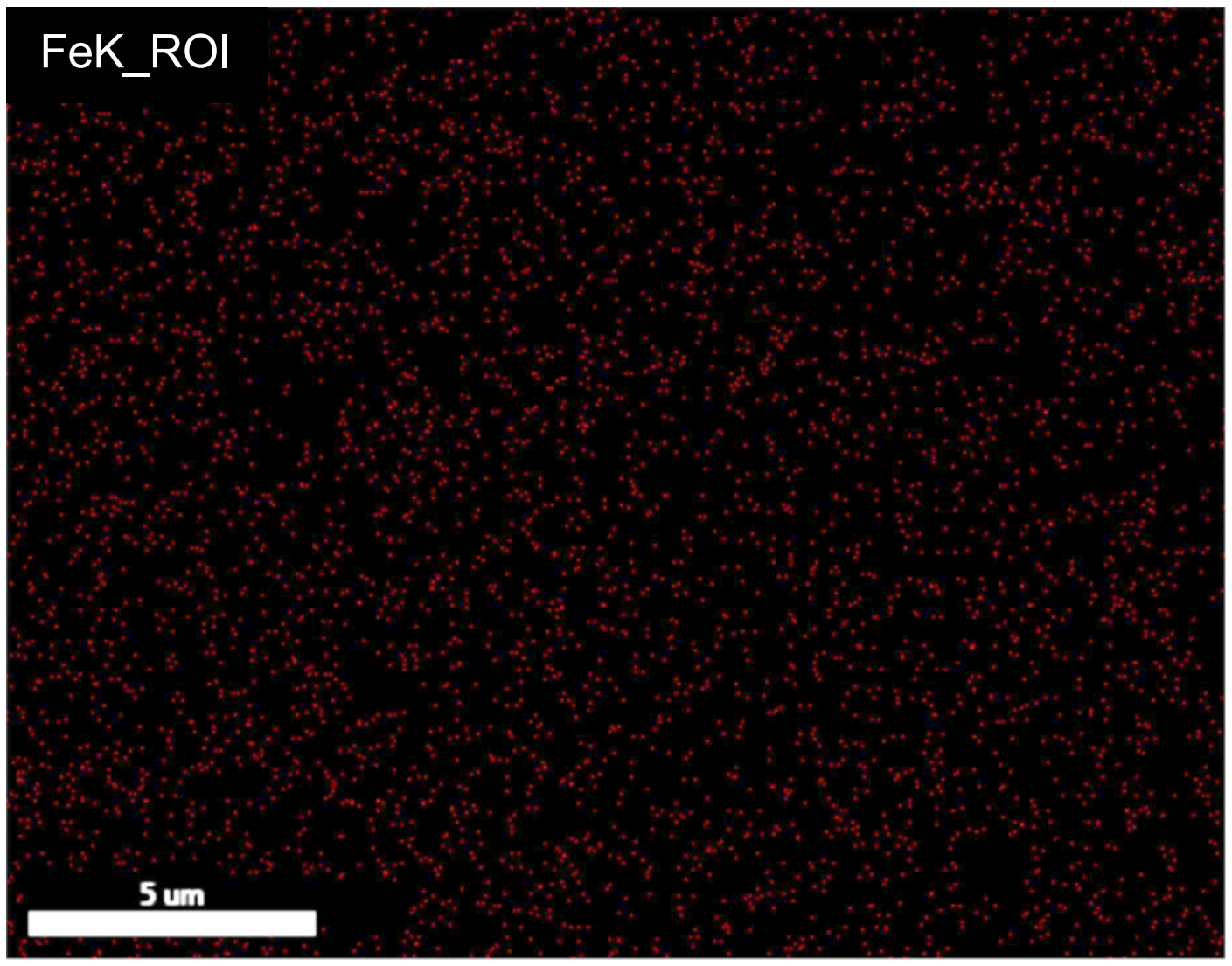

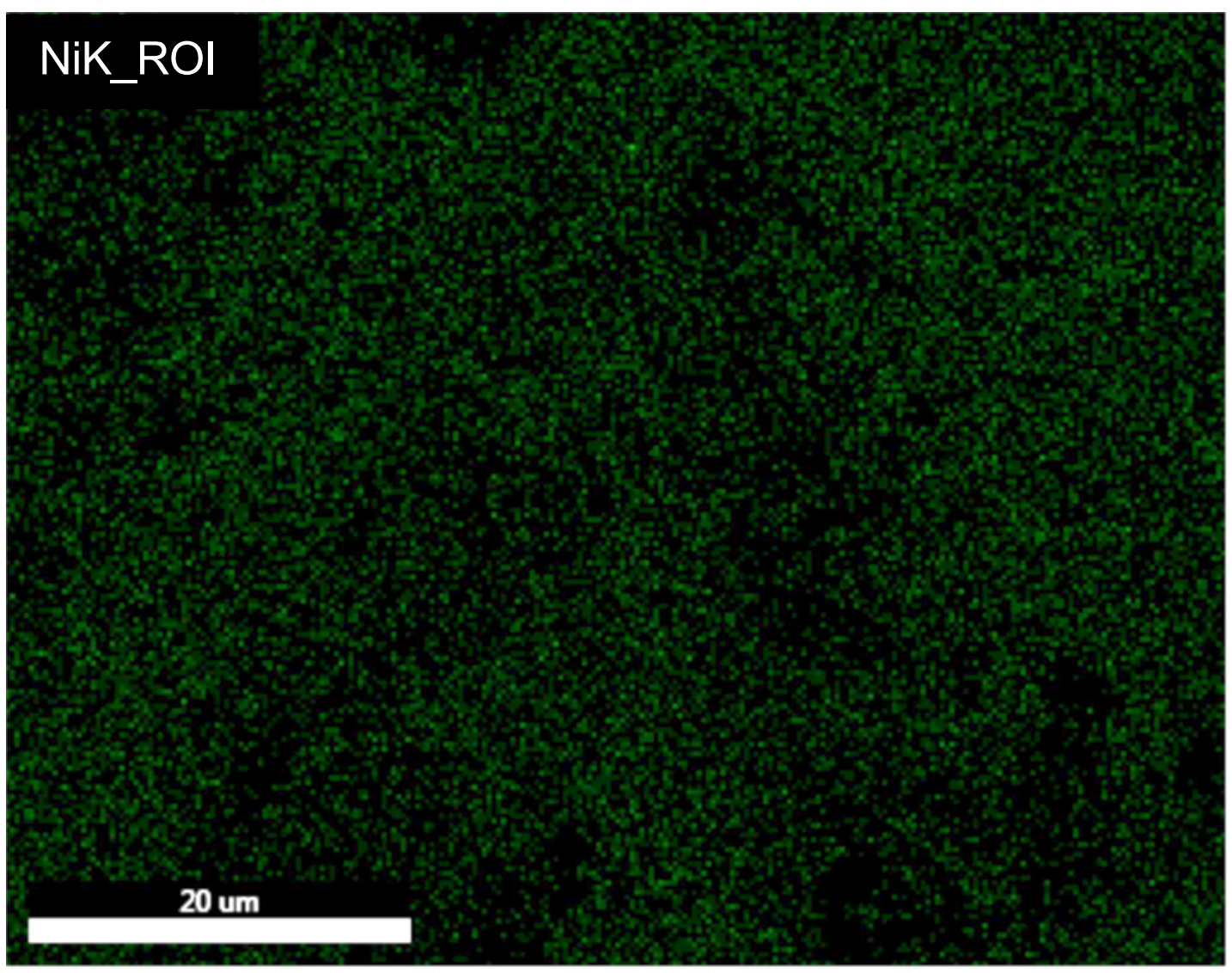

Figure S.6: Elemental mapping images of CNBF sample showing the spatial distribution of Ba, Fe, O, Ce, and Ni throughout the sample.

Characterization

XRD patterns were obtained using a PANalytical X'Pert PRO diffractometer to investigate the crystalline phases of the samples. The surface morphology was examined using a scanning electron microscope with energy dispersive spectroscopy and mapping analysis (SEM/EDS, model Quanta 250 FEG). The functional groups existing in the samples were distinguished through FTIR using a Brucker Vertex 80 V spectrometer in the range of 4000–400 cm^{-1} with a spectral resolution of 2 cm^{-1} to identify the functional groups present in the samples. The optical properties of the samples were rummaged utilizing

UV-Vis diffuse reflectance spectroscopy on a JASCO V-570 spectrophotometer. The band gap energy was defined from the DRS data using the Kubelka-Munk function and Tauc plot analysis, feigning both direct and indirect transitions ($n = 0.5$ and 2, respectively). The Kubelka-Munk function ($F(R)$) is associated with the absorption coefficient (α) and scattering coefficient (s) by the equation $F(R) = (1-R)^2/(2R) = \alpha/s$, where R is the reflectance. The band gap energy (E_g) was calculated from the Tauc plot, which is a plot of $(\alpha h\nu)^n$ versus photon energy ($h\nu$), where α is the absorption coefficient, h Planck's constant, ν the light frequency, and n the exponent associated with the type of electronic transition. Dielectric properties were measured using an LCR-meter (Hitester, model Hioki 3532–50, manufactured in Japan). Magnetic hysteresis loops were measured at room temperature using a vibrating sample magnetometer (VSM, Model LDJ 9600-1) with applied fields up to 10 kOe and with RMS noise floor of 15 nemu at 10 s/point averaging. Saturation magnetization (Ms), coercivity (Hc), retentivity (Mr), and loop area were determined from the hysteresis loops.
